# Supplementary material for: Self-assembled hydrated copper coordination compounds as ionic conductors for room temperature solid-state batteries
Source: Nat Commun. 2024 Feb 5;15:1056. doi: 10.1038/s41467-024-45372-2 (PMC10844207; doi:10.1038/s41467-024-45372-2)
Supplement: Supplementary file 1 — Supplementary Information [file 41467_2024_45372_MOESM1_ESM.pdf]

Supporting information

Self-assembled hydrated copper coordination compounds as ionic conductors for room temperature solid-state batteries

Xiao Zhan[1‡], Miao Li[1‡], Xiaolin Zhao[2‡], Yaning Wang[2‡], Sha Li[1], Weiwei Wang[1], Jiande Lin[1], Zi-Ang Nan[1], Jiawei Yan[1], Zhefei Sun[1], Haodong Liu[3], Fei Wang[4], Jiayu Wan[5], Jianjun Liu[2*], Qiaobao Zhang[1,6*] and Li Zhang[1*]

[1]State Key Laboratory of Physical Chemistry of Solid Surfaces, College of Chemistry and Chemical Engineering, College of Materials, Tan Kah Kee Innovation Laboratory, Collaborative Innovation Center of Chemistry for Energy Materials, Xiamen University, Xiamen 361005, Fujian, China.
E-mail: zhangqiaobao@xmu.edu.cn, zhangli81@xmu.edu.cn
[2]State Key Laboratory of High Performance Ceramics and Superfine Microstructures, Shanghai Institute of Ceramics, Chinese Academy of Sciences, Shanghai 200050, China.
E-mail: jliu@mail.sic.ac.cn
[3]Chemical Engineering, UC San Diego, La Jolla, CA 92093, USA
[4]Department of Materials Science, Fudan University, Shanghai, 200433 China
[5]Future Battery Research Center, Global Institute of Future Technology, Shanghai Jiaotong University, Shanghai 200240, China
[6]Shenzhen Research Institute of Xiamen University, Shenzhen 518000, China

Supplemental Figures and Tables

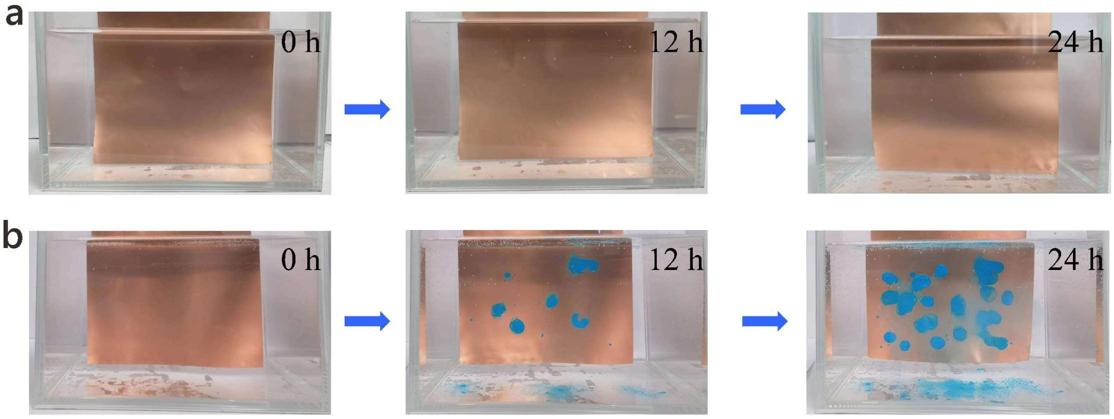

Figure S1. Digital photographs of the production speed of CuMH powders at different reaction times a) without and b) with the addition of $LiNO_3$. The production speed of CuMH has been greatly accelerated with the aid of $LiNO_3$, while the reaction rate without $LiNO_3$ is extremely low.

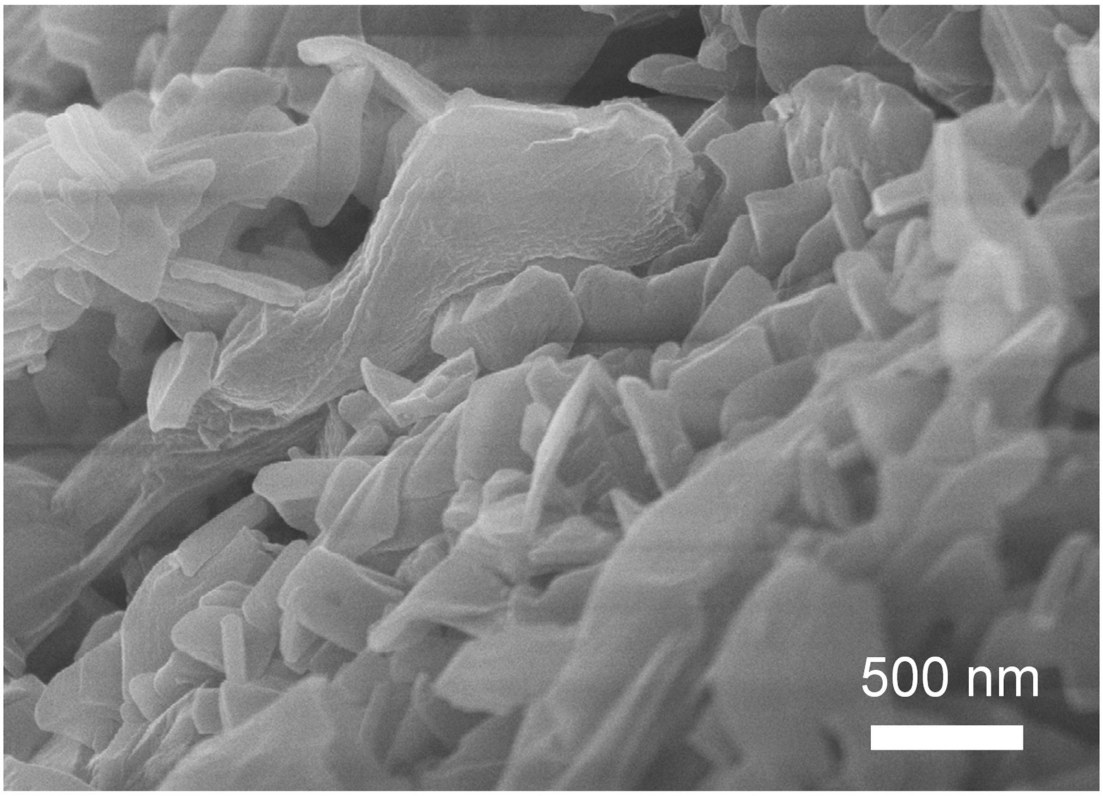

Figure S2. SEM image of irregular CuMH nanosheets obtained from the traditional solvothermal method using $Cu(NO_3)_2$ as the precursor.

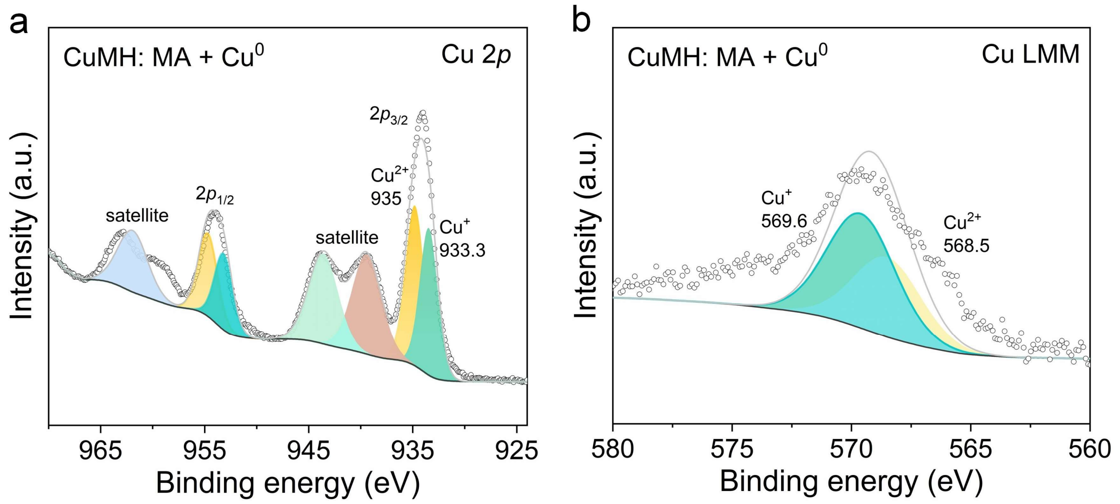

Figure S3. a) Cu 2*p* XPS and b) Cu Auger LMM spectra of CuMH film prepared from MA and Cu^0 foil. The raw data and fitted data plots are shown as gray hollow points and grey solid lines, respectively.

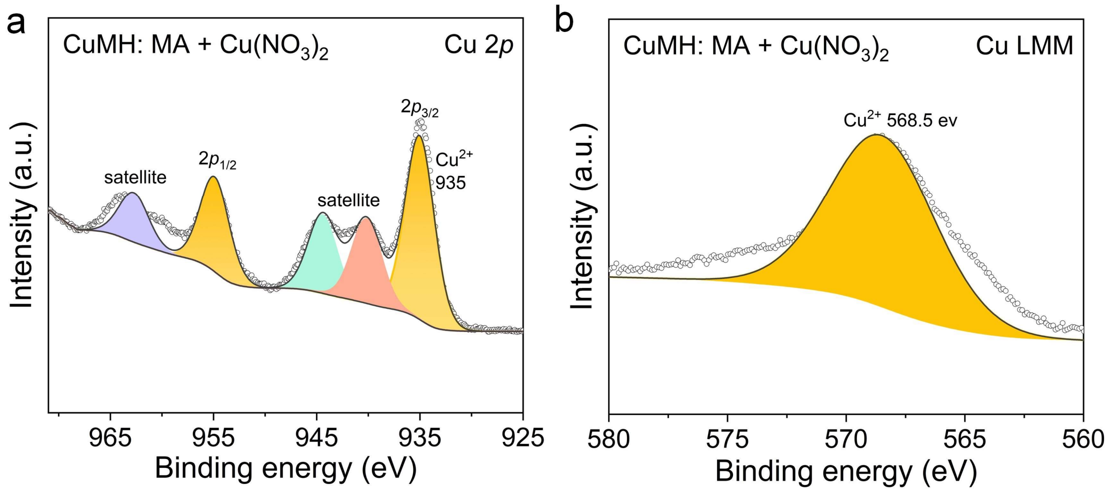

Figure S4. a) Cu 2*p* XPS and b) Cu Auger LMM spectra of CuMH film prepared from MA and $Cu(NO_3)_2$ salts. The raw data and fitted data plots are shown as gray hollow points and grey solid lines, respectively.

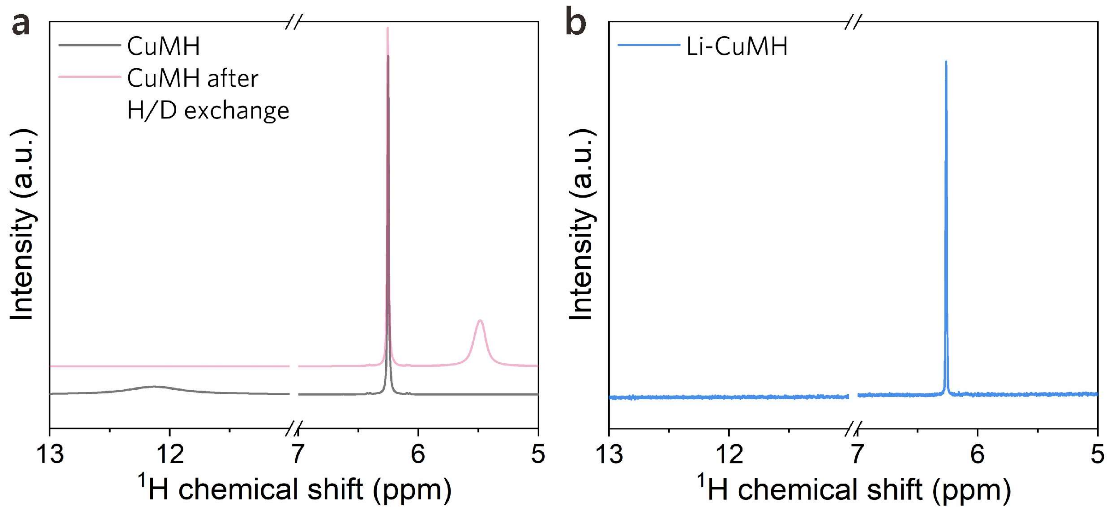

Figure S5. a) ^{1}H NMR spectra of original CuMH sample and the CuMH sample after hydrogen/deuterium (H/D) exchange. b) ^{1}H NMR spectra of the Li-CuMH sample.

As shown in Fig. S5a, the characteristic peaks in the ^{1}H NMR spectrum of the pristine CuMH sample are assigned to resonances of hydroxyl hydrogens (-COOH)[1] and the methylene protons (-CH=CH-)[2], respectively. After the hydrogen/deuterium (H/D) exchange reaction, the peaks appearing at 6.2 and 5.5 ppm are identified to resonances of the methylene protons (-CH=CH-) and protons of HOD, while the peak at 12.1 ppm completely disappears, convincingly demonstrating the existence of hydroxyl hydrogen (-COOH) in the CuMH sample. Moreover, the ^{1}H NMR spectrum of the Li-CuMH sample only shows one characteristic peak of the methylene protons (-CH=CH-) at 6.2 ppm, since the H^{+} ions on the carboxylic acid of CuMH underwent an ion-exchange reaction with the Li^{+} ions during the soaking process (Fig. S5b).

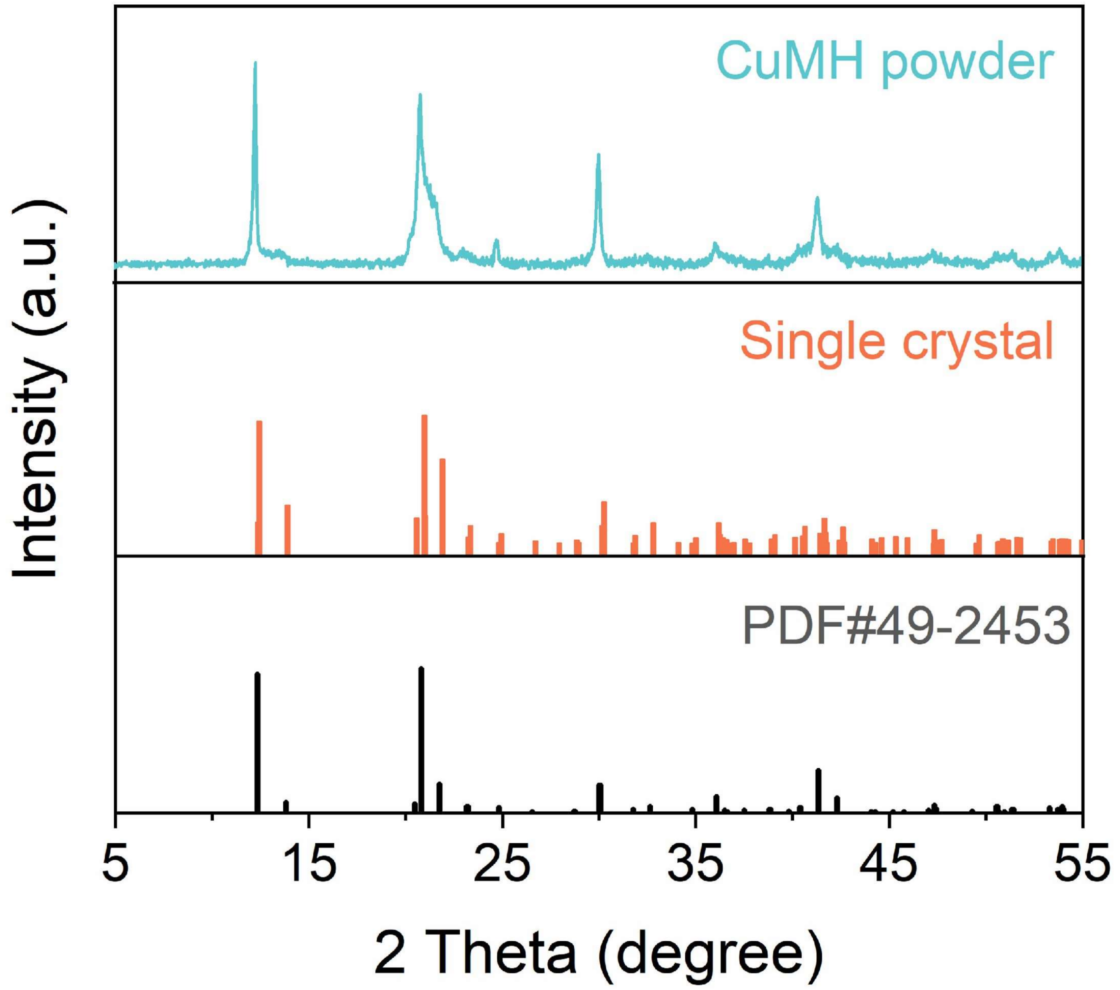

Figure S6. Powder XRD pattern of the as-obtained CuMH powder and single crystal XRD pattern of sample grown in water and the corresponding standard powder diffraction card (PDF#49-2453).

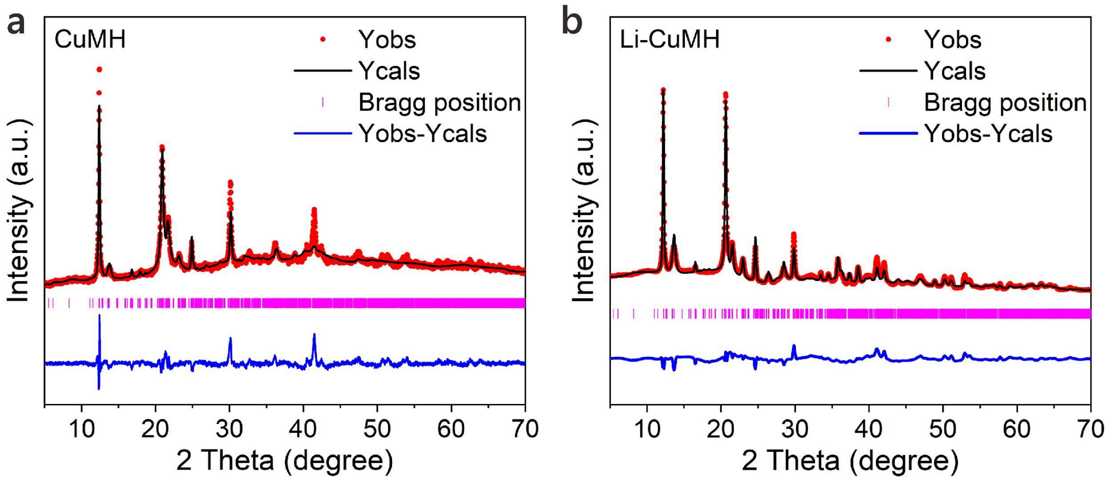

Figure S7. Rietveld refinement patterns of XRD for the $Cu^{I/II}$MH (a) and Li-$Cu^{I/II}$MH (b) samples. The observed and calculated intensities are shown as the red circles and the black solid line, respectively. The bottom blue line exhibits the fitting residual difference. The Bragg positions are represented by purple scale lines.

Table S1. Structural analysis results obtained from Rietveld refinement XRD patterns of the $Cu^{I/II}$MH and Li-$Cu^{I/II}$MH samples.

Samples	Space group	*a*/ Å	*b*/ Å	*c*/ Å	V/ $Å^3$	*α*/°	*β*/°	*γ*/°	R_{wp} (%)	R_p (%)
$Cu^{I/II}$MH	P1	15.512	15.857	7.713	1750.552	90.735	112.660	89.583	7.45	5.24
Li-$Cu^{I/II}$MH	P1	15.574	16.023	7.759	1792.155	90.070	112.240	89.980	8.43	6.50

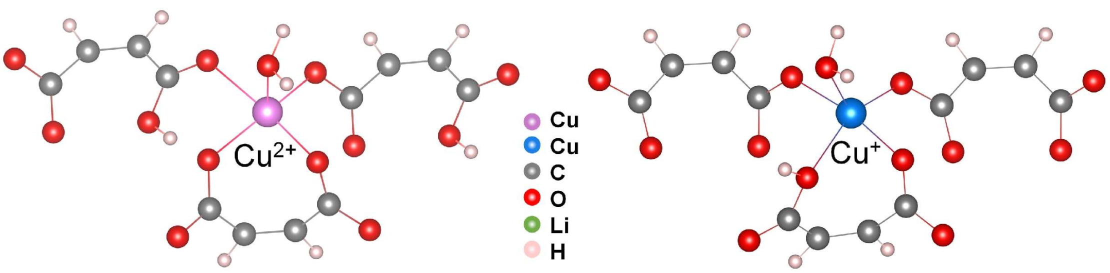

Figure S8. Local structures of Cu^+ and Cu^{2+} coordinated with maleic acid in the CuMH crystal, respectively.

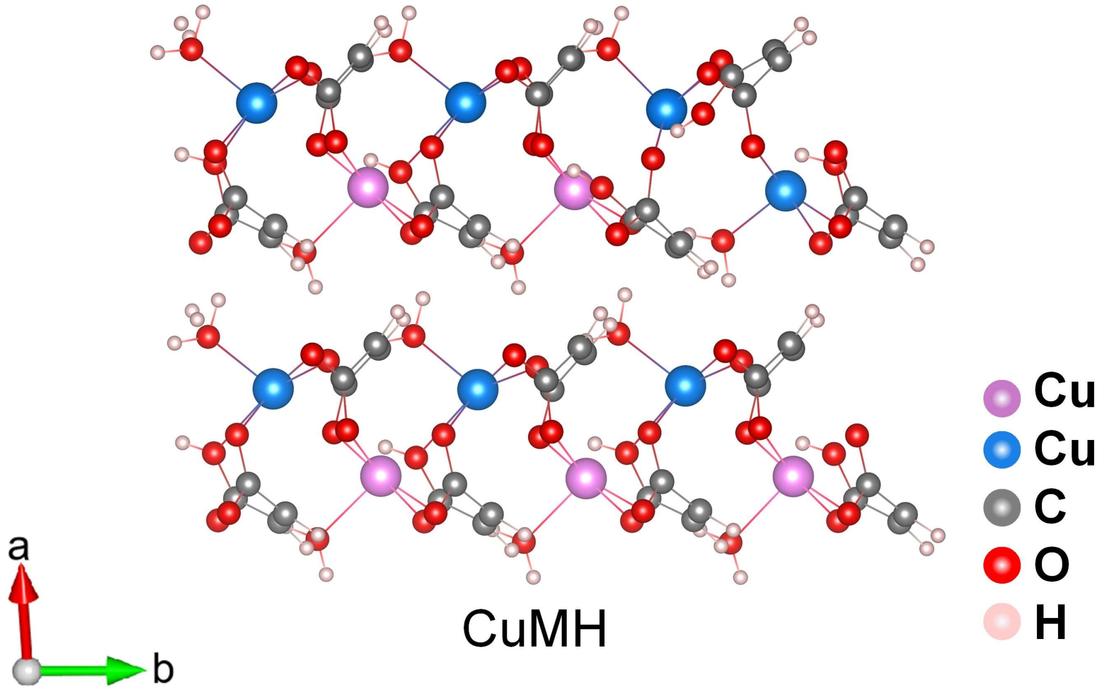

Figure S9. The crystal structure of CuMH containing Cu^{+} and Cu^{2+} along the c-axis.

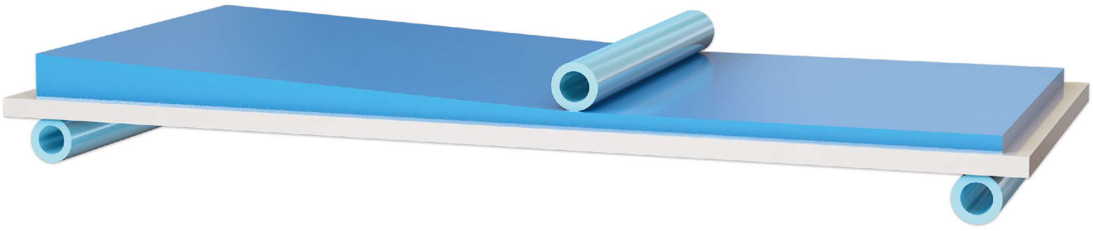

Figure S10. Schematic diagram of a roller press for rolling the CuMH-PTFE paste into compact film.

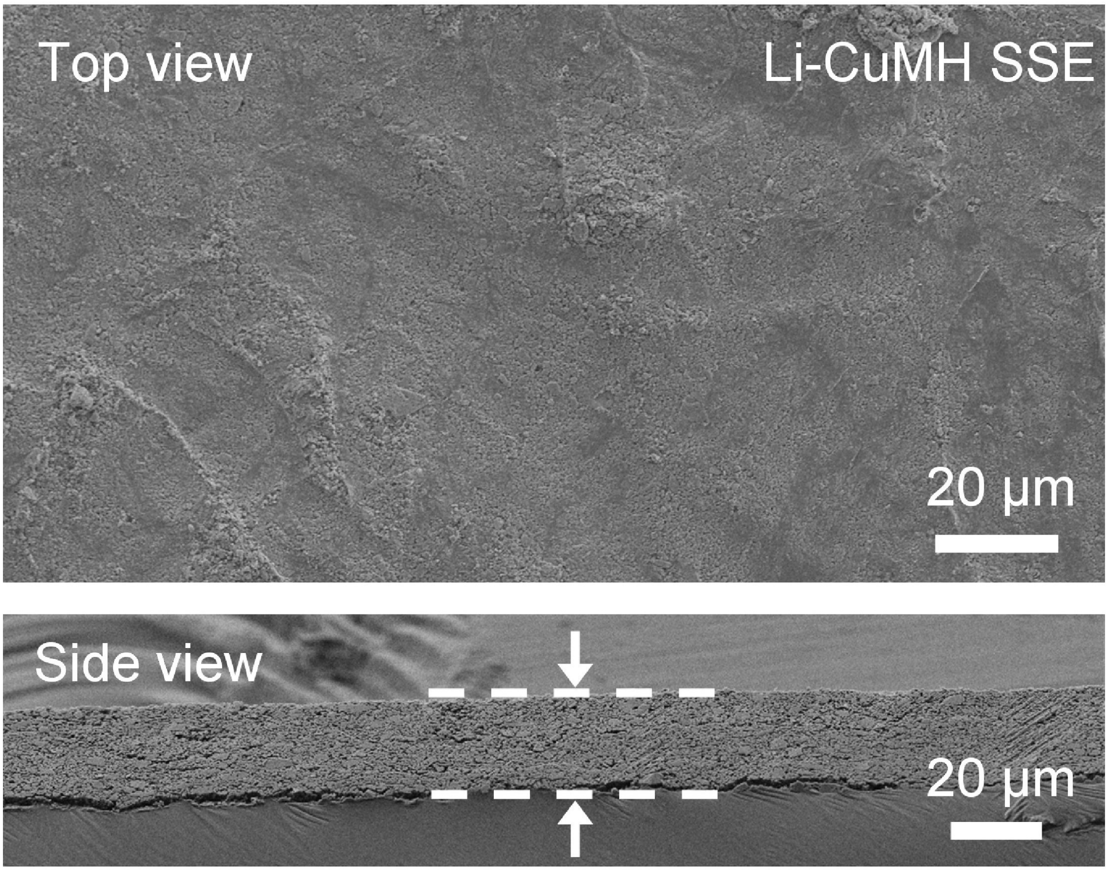

Figure S11. Top view and side view SEM images of the Li-CuMH SSE film.

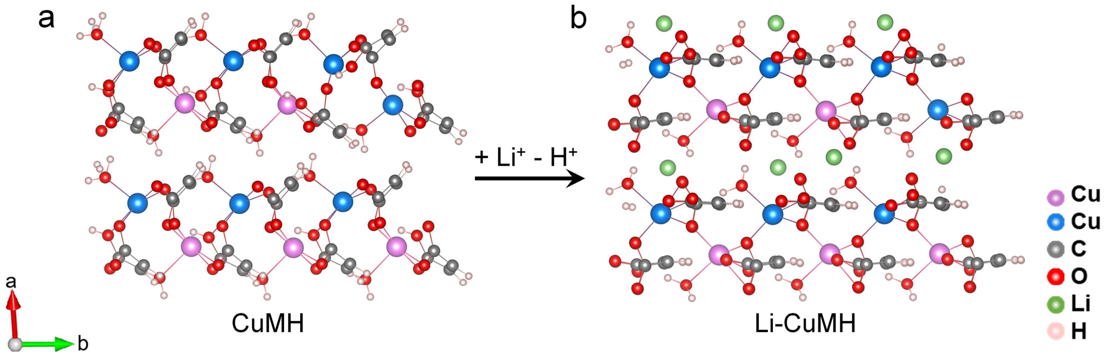

Figure S12. The crystal structure of Li-CuMH produced by the ion-exchange reaction between Li^+ ions and H^+ from CuMH. a) The crystal structure of CuMH. b) The crystal structure of Li-CuMH.

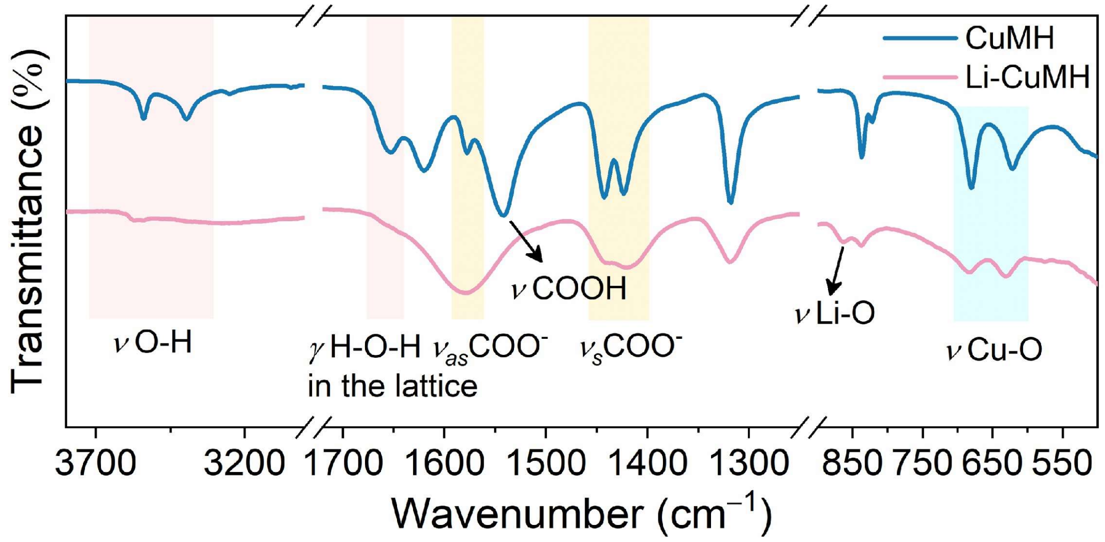

Figure S13. FT-IR spectra of the CuMH and Li-CuMH samples.

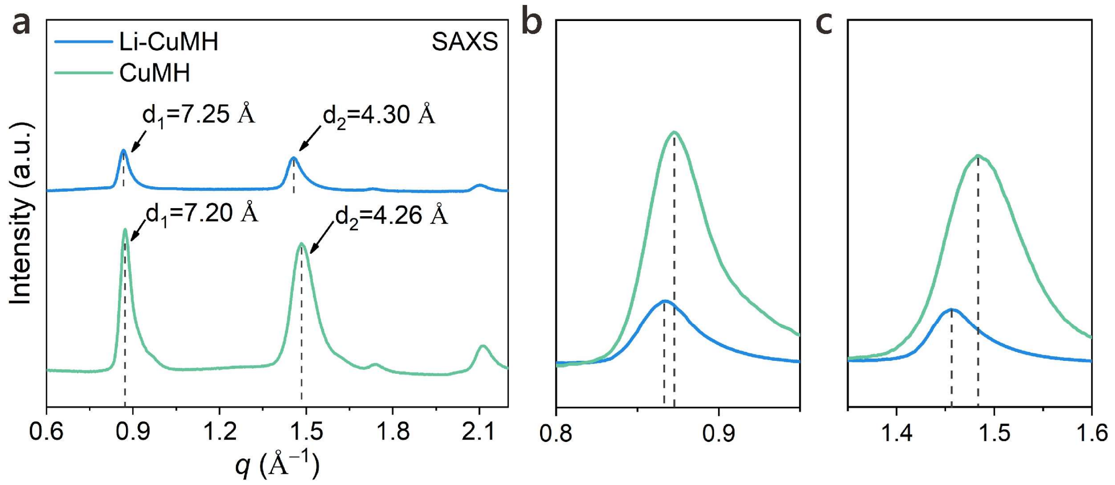

Figure S14. The SAXS curves a) of CuMH and Li-CuMH, and the corresponding zoom-in curves in the q range of b) 0.8-0.95 Å and c) 1.35-1.6 Å, respectively. The corrected scattering intensity was plotted relative to the scattering vector q.

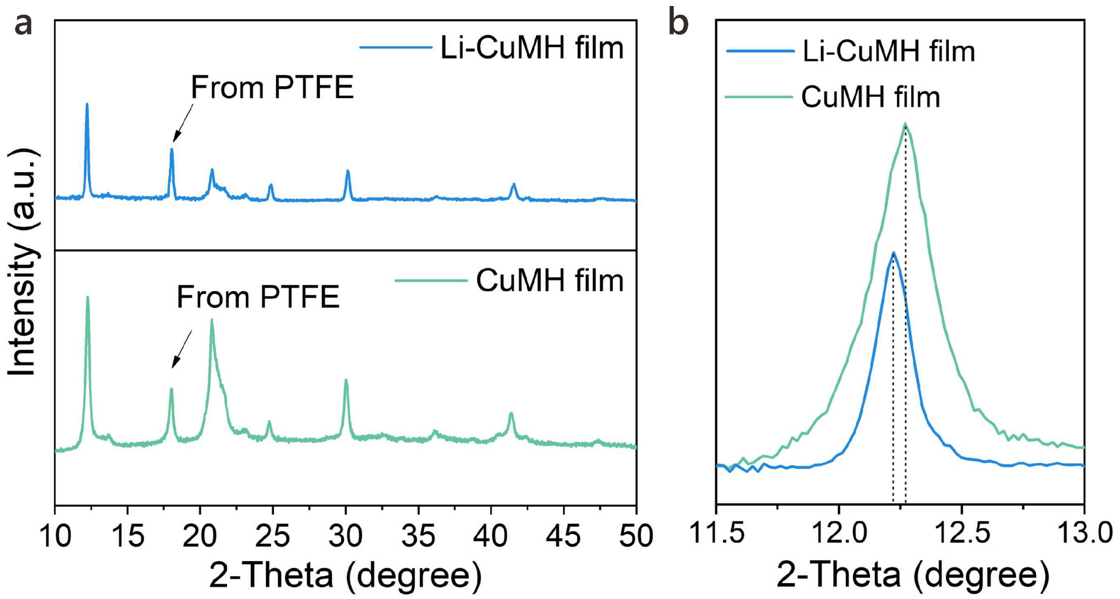

Figure S15. a) XRD patterns of CuMH film and Li-CuMH SSE film and b) the corresponding enlarged spectra in the 2-theta range of 11.5° and 13°.

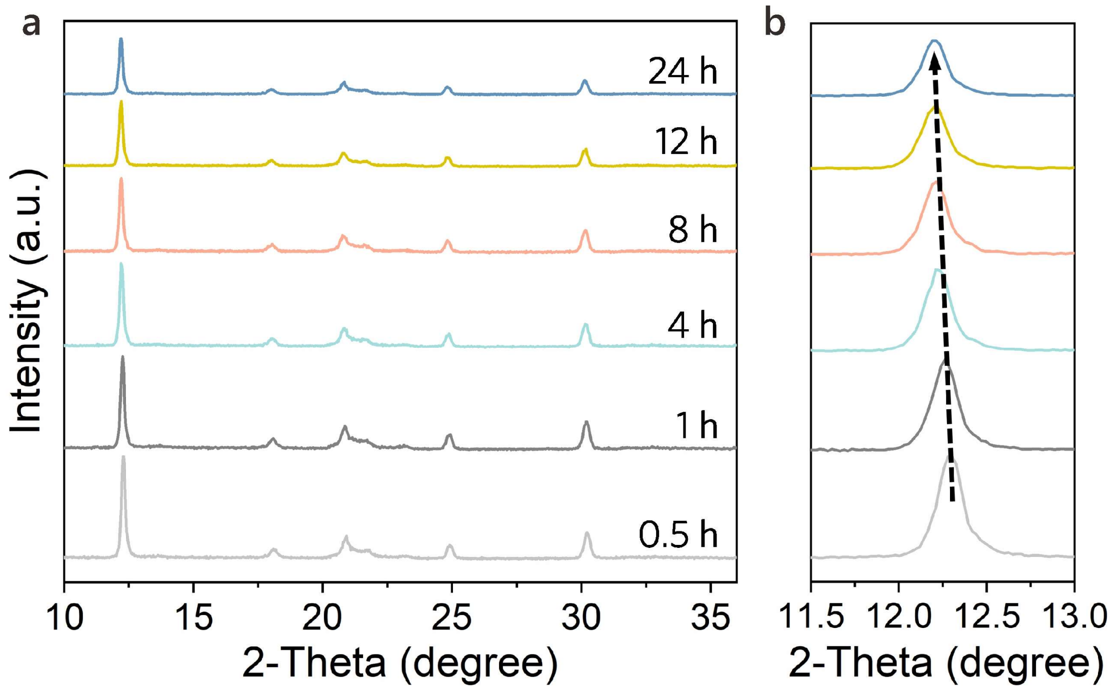

Figure S16. a) XRD patterns of Li-CuMH SSE films after soaking in the non-aqueous electrolyte containing LiTFSI salts for different times and b) the corresponding enlarged spectra in the 2-theta range of 11.5° and 13°.

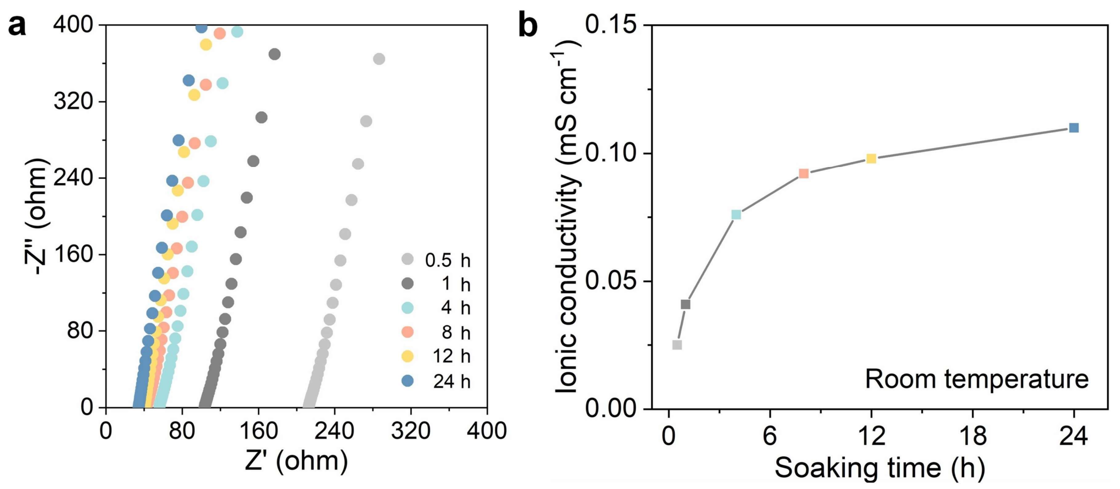

Figure S17. a) Nyquist plots of Li-CuMH SSE films with different Li^+ implantation times and b) the corresponding ionic conductivities at room temperature.

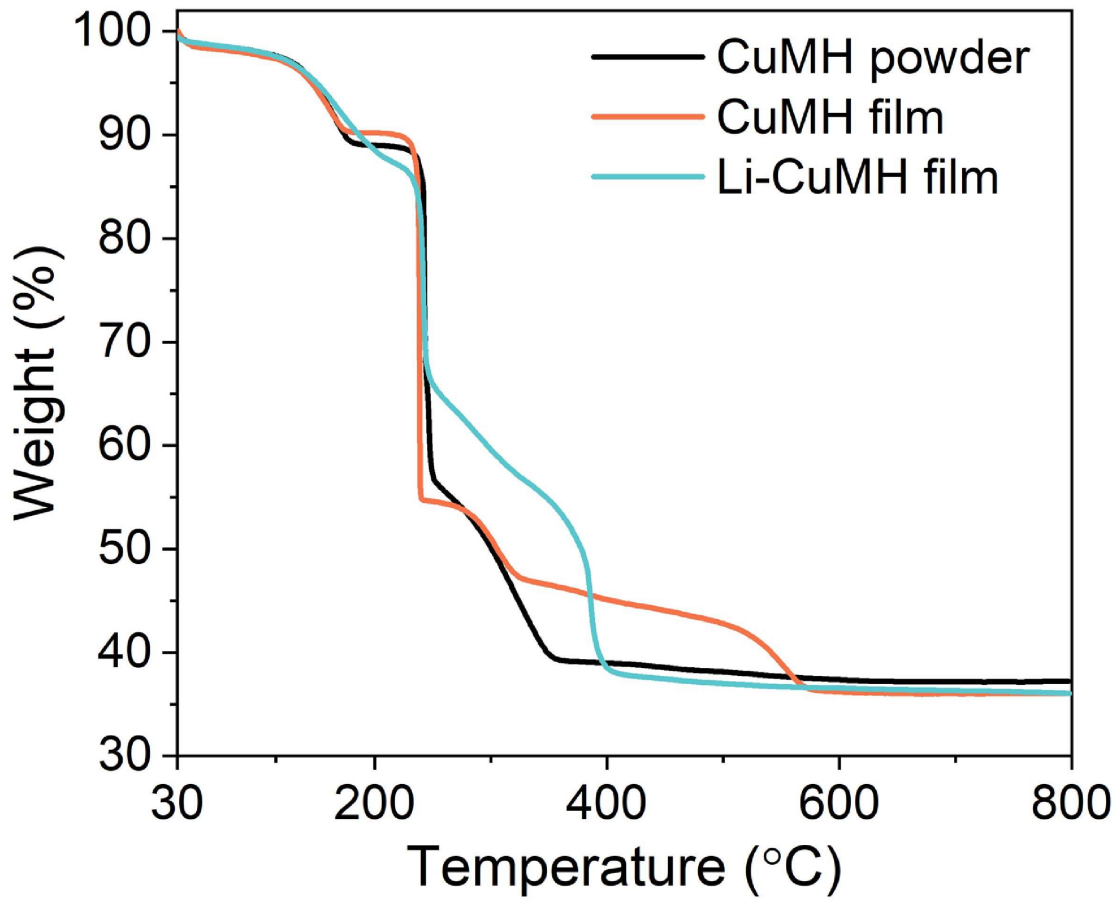

Figure S18. TGA curves of CuMH powder, CuMH film and Li-CuMH film. All samples remained stable until the temperature reaches 100 °C, and then gradually dehydrated in the temperature range of 100-160 °C. This directly demonstrates that the

water in the CuMH and Li-CuMH samples is in the structural state, rather than free water.

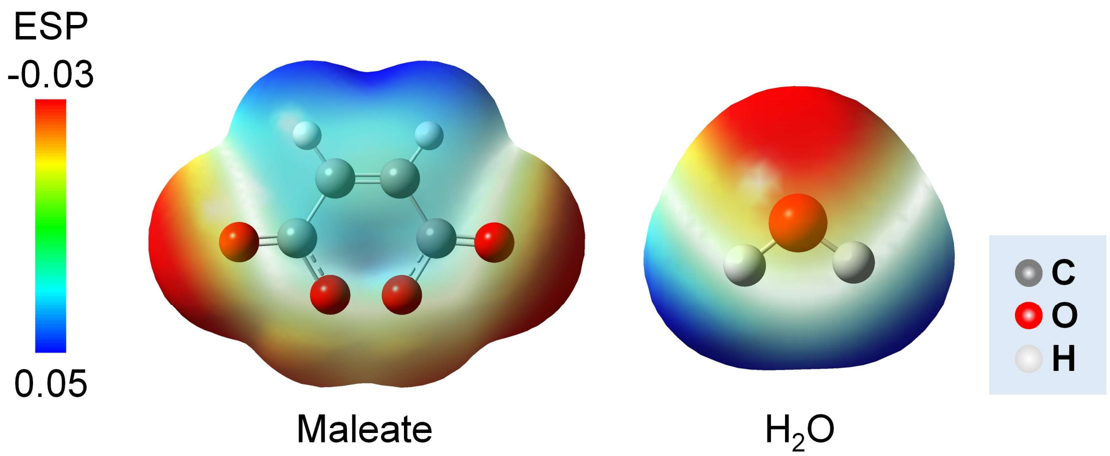

Figure S19. Electrostatic potential (ESP) distribution of maleate unit and structural water in Li-CuMH.

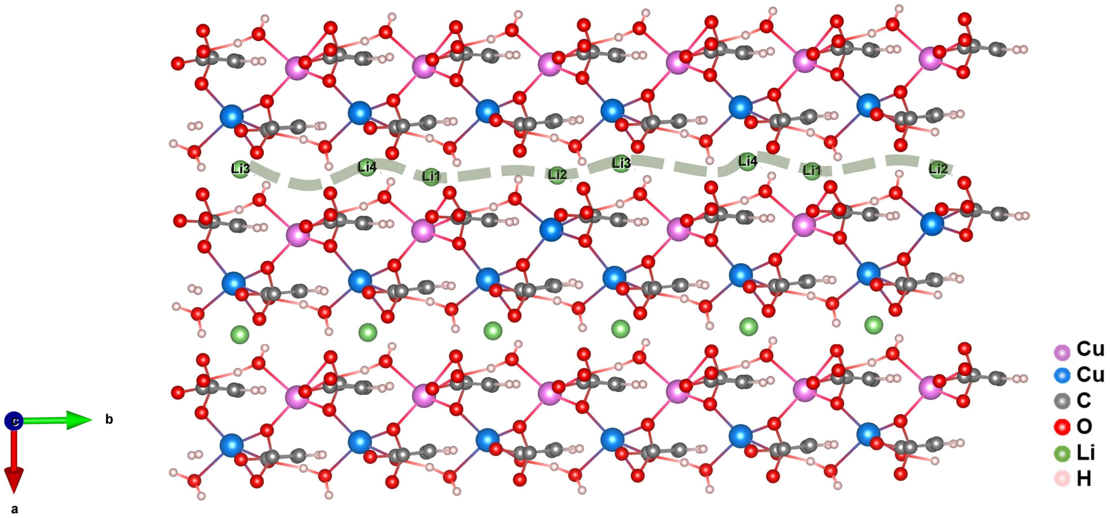

Figure S20. 1D Li^+ ion migration path in the direction of the [010] chain along the *b*-axis (green dotted line).

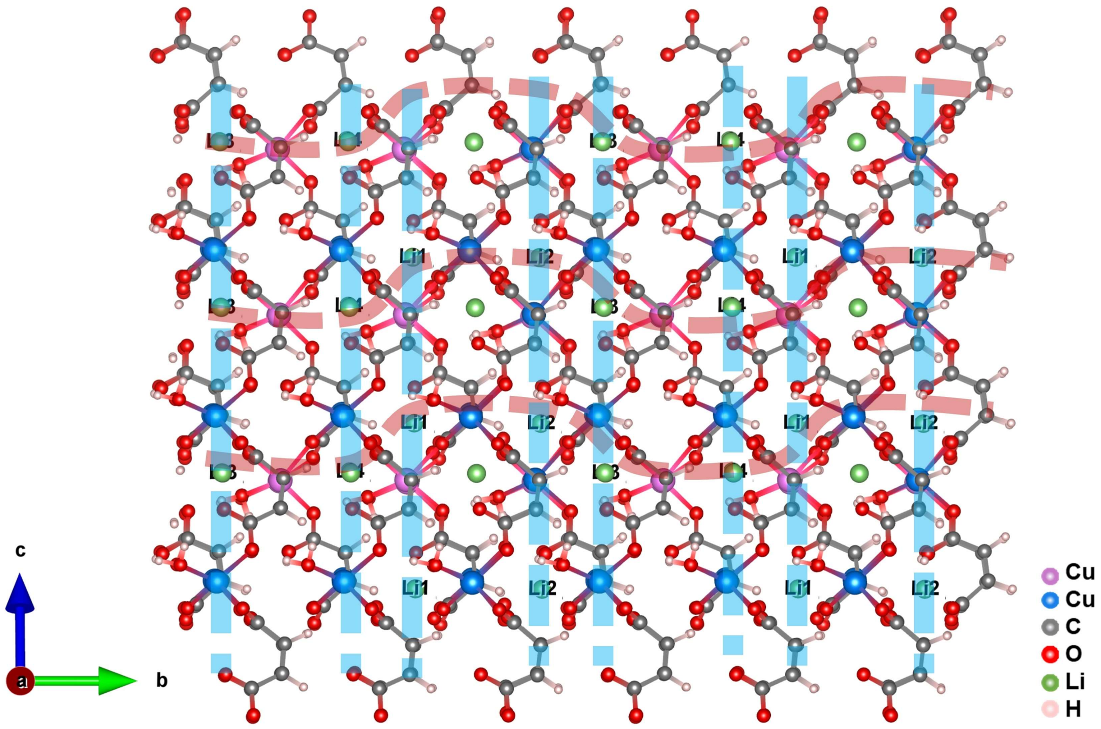

Figure S21. 2D Li^+ ion migration path in the *bc* plane.

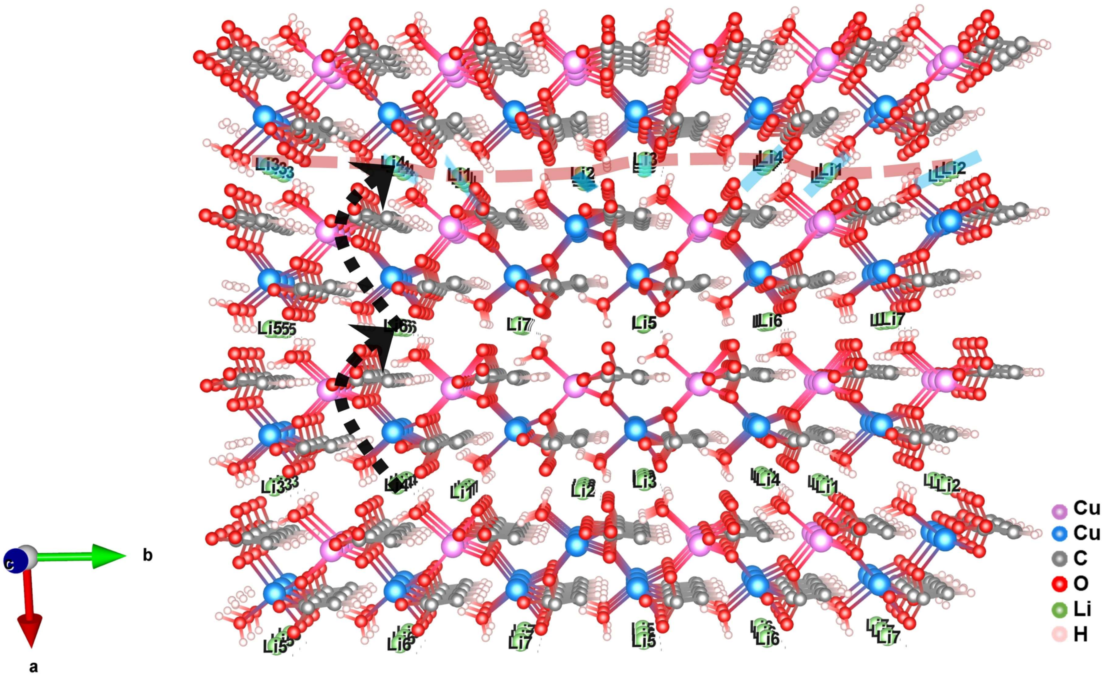

Figure S22. 3D Li^+ ion migration path.

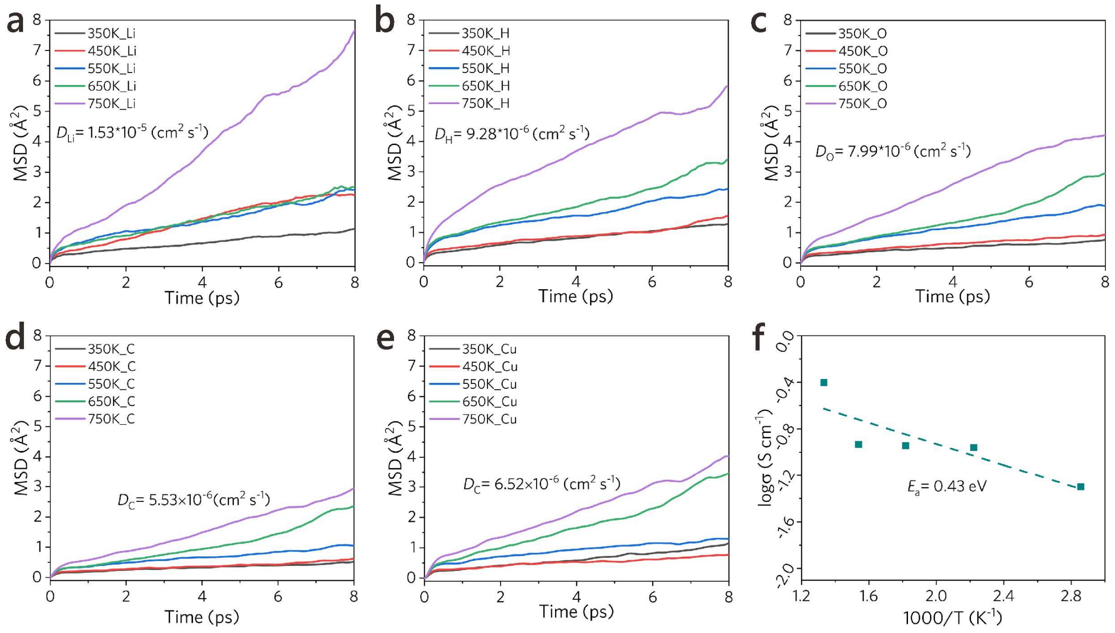

Figure S23. Mean square displacement (MSD) plots of a) Li, b) H, c) O d) C and e) Cu in Li-CuMH at 350, 450, 550, 650 and 750 K, respectively. f) Arrhenius plots plotted according to (a-e).

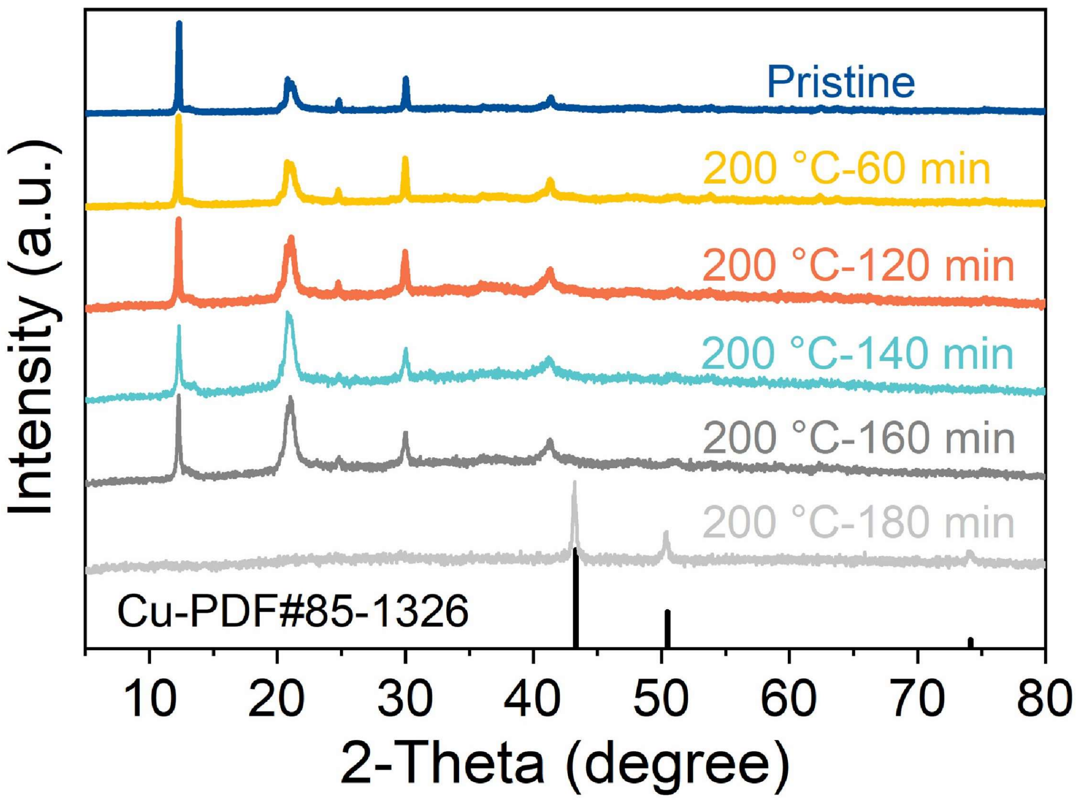

Figure S24. XRD patterns of CuMH powders after sintering at 200 °C for different times.

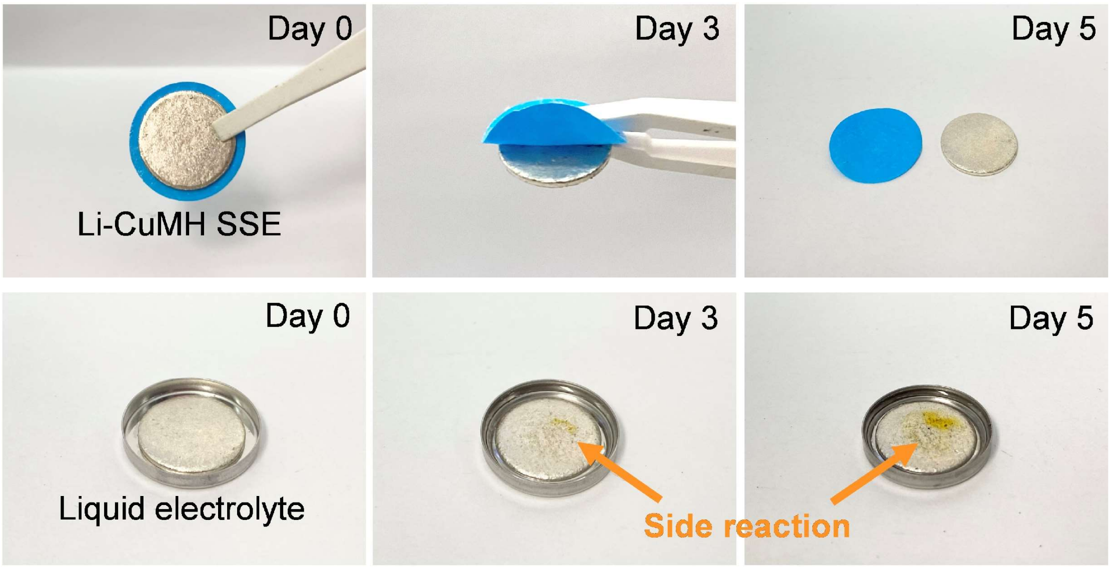

Figure S25. Digital images of chemical stability and side reactions of Li metal electrodes after contact with Li-CuMH SSE and organic liquid electrolyte for different times.

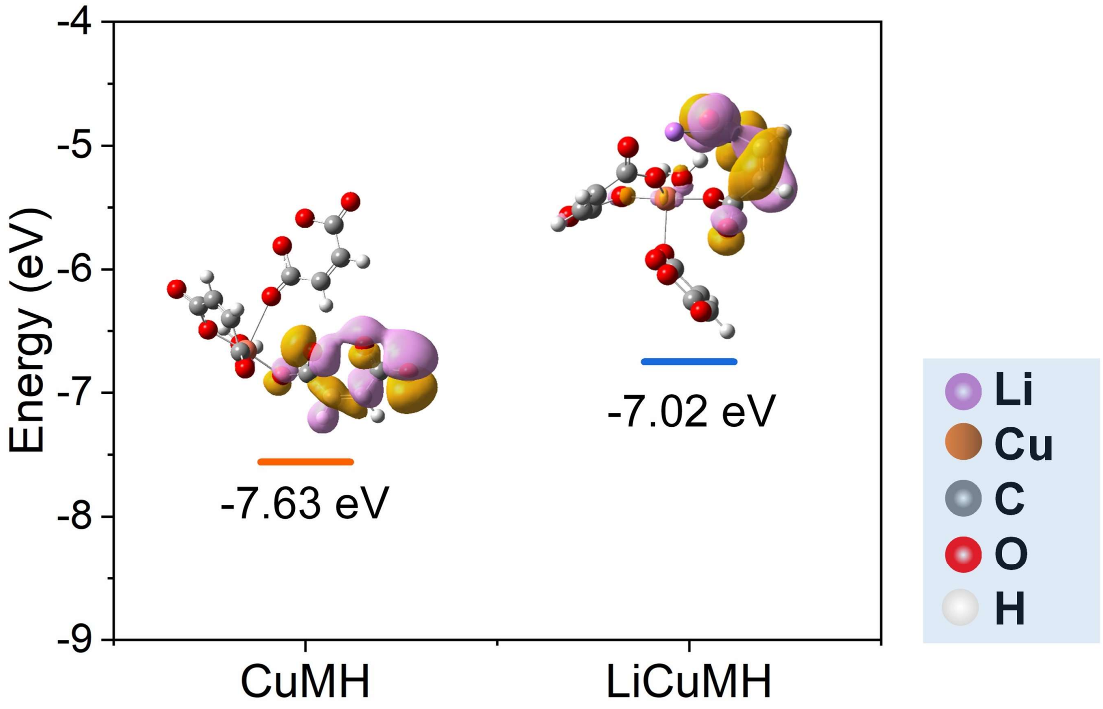

Figure S26. HOMO energies of CuMH and Li-CuMH.

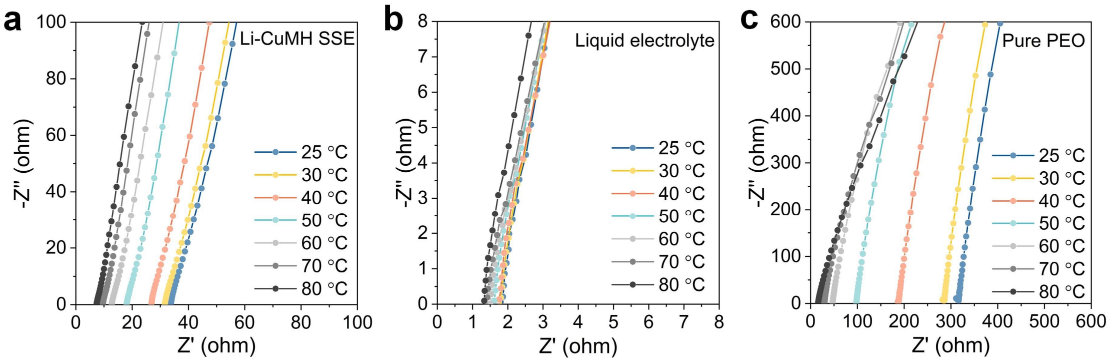

Figure S27. Nyquist plots of a) Li-CuMH SSE, b) liquid electrolyte and c) pure PEO electrolyte from 25 to 80 °C.

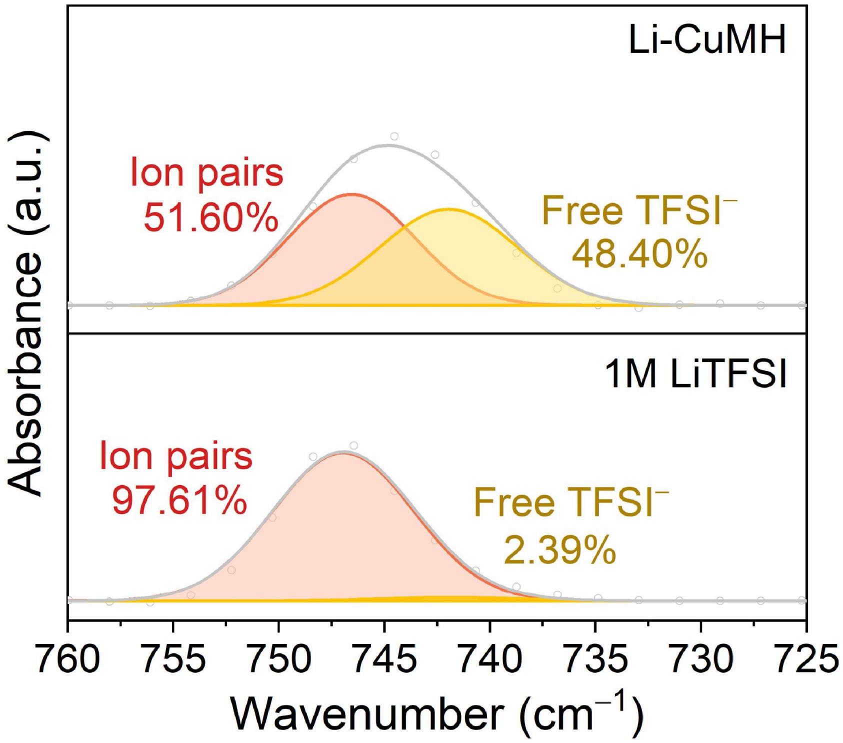

Figure S28. FT-IR spectra in the region of TFSI⁻ vibration of 1M LiTFSI in DOL/DME liquid electrolyte and Li-CuMH SSE.

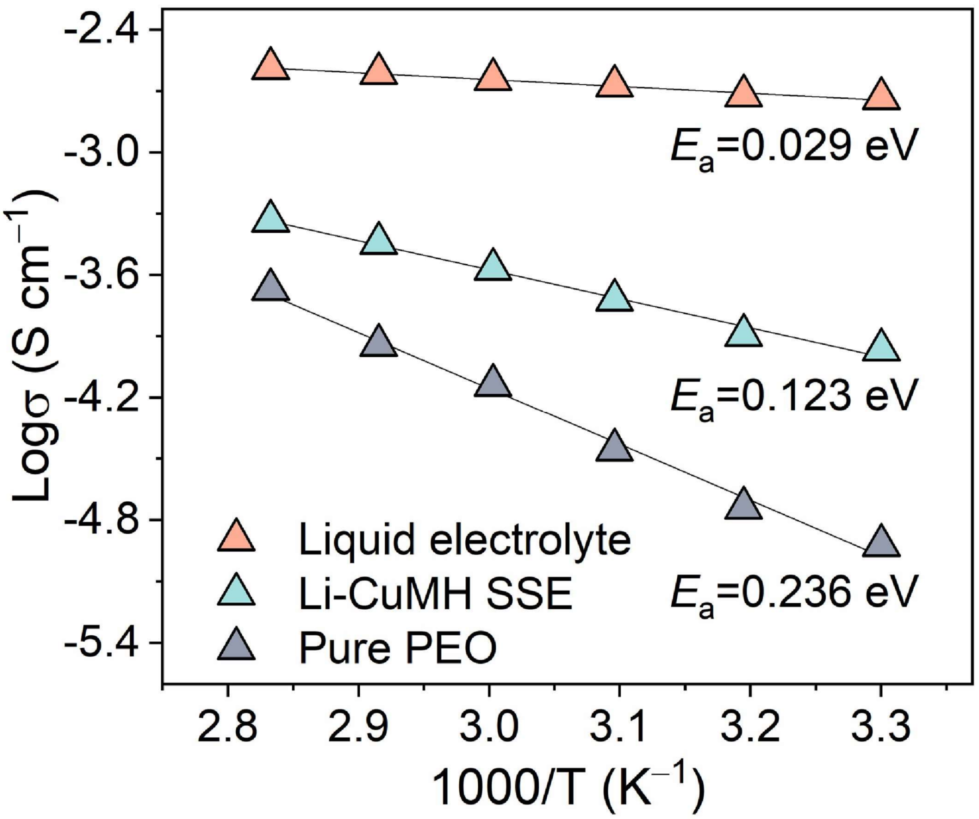

Figure S29. Arrhenius plots of the organic liquid electrolyte, Li-CuMH SSE and solid polymer PEO electrolyte.

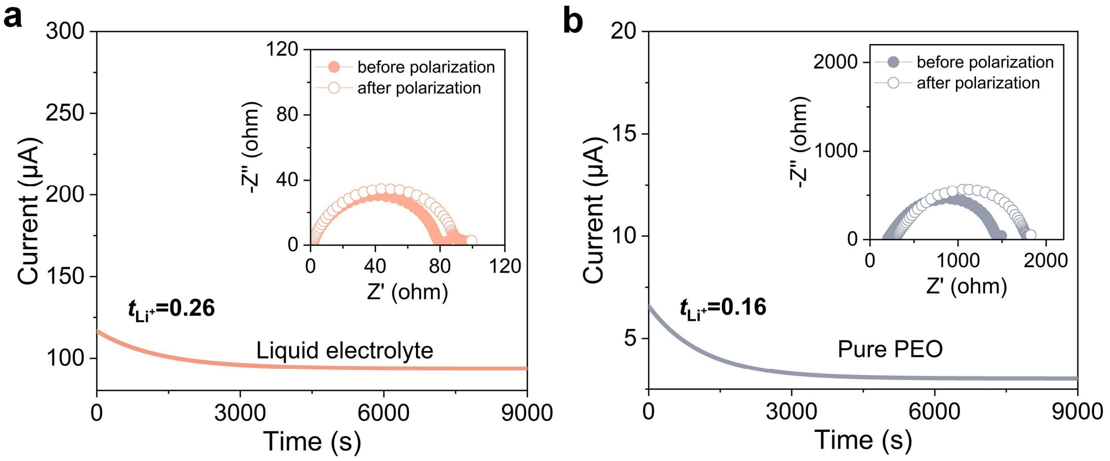

Figure S30. Li^+ transference number measurement of a) the organic liquid electrolyte and b) solid polymer PEO electrolyte. The insets are the Nyquist plots of Li/liquid electrolyte/Li and Li/PEO electrolyte/Li symmetric cells before and after polarization, respectively.

Supplementary Note: The Li^+ transference number (t_{Li+}) was measured on Li/Li-CuMH SSE/Li symmetric cells by the chronoamperometry test. The Li^+ transference number were calculated by the equation (2):

$$t_{Li^+} = \frac{I_{SS}}{I_O} \times \frac{V - I_O R_O}{V - I_{SS} R_{SS}}$$

where V, I_O, I_{SS}, R_O and R_{SS} represent the applied voltage (10 mV), the initial and steady-state currents and the impedance before and after polarization, respectively. As show in Figure S30a, I_O, I_{SS}, R_O and R_{SS} are 120 μA, 93μA, 78 ohms and 87 ohms, respectively. Thus, the Li^+ transference number of the liquid electrolyte is 0.26. Similarly, the Li^+ transference number of the PEO electrolyte is calculated as 0.16.

Table S2. The parameters measured by *i-t* curves and EIS for calculating the Li^+ transference number. This table refers to Figs. 3g and S30.

	ΔV (mV)	I_o (μA）	I_{ss} (μA）	R_o (ohm)	R_{ss} (ohm)	t_{Li+}
Li-CuMH	10	19.476	16.276	267	295	0.77
Liquid electrolyte	10	120	93	78	87	0.26
Pure PEO	10	6.578	3.020	1275	1552	0.16

I_o and I_{ss} are initial and stable current (μA) during polarization. R_o and R_{ss} are the impedance (ohm) before and after polarization.

Table S3. Li/Li symmetric cell performance of Li-CuMH SSE and SSEs in the literatures. This table refers to Figure 4b.

Electrolyte	Type	Current density (mA cm^{-2})	Areal Capacity (mAh cm^{-2})	Overpotential (mV)	Ref.
Li-Cu-CNF	Cellulose	0.5 (RT)	1	~100	[3]

21-*β*-CD-*g*-PTFEMA	Topological polymer	0.1 (70 °C)	0.1	100	[4]
LIMIC-15	Rigid-rod polymer	0.2 (22 °C)	0.1	~200	[5]
PEO/$Mg(ClO_4)_2$	PEO-based	0.4 (55 °C)	0.1	~300	[6]
PEO/Li_2S_6	PEO-based	0.2 (40 °C)	0.1	~300	[7]
HKUST-1	MOF-based	0.125 (25 °C)	0.25	~20	[8]
CD-COF-Li	COF-based	0.2 (RT)	0.2	40	[9]
Li-CuMH SSE	2D lamellar	0.9 (25 °C)	0.45	284	This work

RT: room temperature.

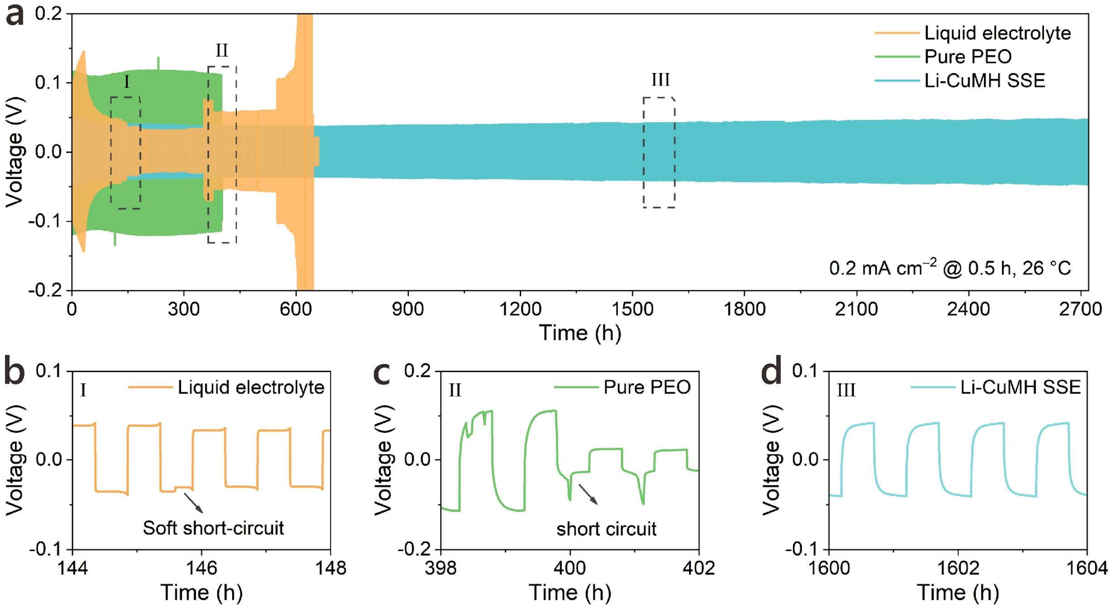

Figure S31. a) Voltage profiles of the Li/Li symmetric cells assembled with liquid electrolyte (1M LiTFSI in DOL/DME), PEO electrolyte and Li-CuMH SSE at the current density of 0.2 mA cm^{-2} and areal capacity of 0.1 mAh cm^{-2}. b-d) The enlarged voltage profiles highlighted in the I, II and III regions of a).

As Fig. S31 demonstrates, the Li/Li-CuMH SSE/Li battery can stably cycle for more than 2700 h without short circuit at 0.2 mA cm^{-2} (0.1 mAh cm^{-2}) and room temperature. Under the same test conditions, the Li/liquid electrolyte/Li (Li/LE/Li) cell began to exhibit instability only after 146 h, and the overpotential gradually decreased. The polarization of the Li/LE/Li cell suddenly increased after 350 h, indicating that the dendritic Li growth caused a short circuit in the battery. The Li/pure PEO/Li symmetric cell was tested under the same conditions but at a higher temperature of 60 °C. As expected, the battery short-circuited after only 400 h owing to the sacrificial mechanical strength at high operating temperatures. More importantly, the Li/Li-CuMH SSE/Li battery showed a low overpotential of below 50 mV during the whole stripping/plating process at room temperature. These results strongly show that the Li-CuMH SSE has excellent dendrite-inhibition ability and outstanding electrochemical stability.

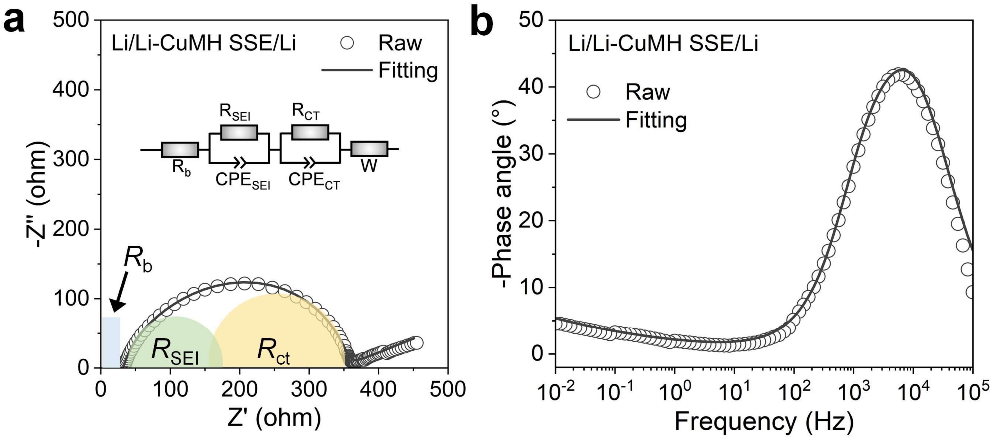

Figure S32. a) Nyquist plot and b) impedance phase angles versus frequencies plot of the Li/Li-CuMH SSE/Li symmetric cell before cycling at room temperature ranging from 10^5 to 10^{-2} Hz. The inset in a) is the corresponding equivalent circuit used to fit the Nyquist plot, in which R_b represents the bulk resistance, R_{SEI} is the SEI resistance, R_{CT} stands for charge-transfer resistance, CPE_{SEI} is the capacitance of SEI, CPE_{CT} is the capacitance describing the electrical double layer at the SSE/Li interface, and W is the Warburg impedance (linear line).

Table S4. The values of R_b, R_{SEI}, R_{ct}, and $R_{SSE/Li}$ obtained by fitting each individual spectrum of Li/Li-CuMH SSE/Li symmetric cells after various cycle numbers at 0.5 mA cm^{-2}.

Cycle number	R_b (ohm)	R_{SEI} (ohm)	R_{ct} (ohm)	$R_{SSE/Li}$=R_{SEI} + R_{ct} (ohm)
0th	30.67	92.30	221.9	314.20
1st	32.10	41.58	284.1	325.68
5th	31.50	108.9	239.0	347.90
10th	33.25	190.9	201.4	392.30
20th	32.92	179.8	209.7	389.50
40th	32.62	109.9	297.0	406.50
60th	32.68	70.50	316.2	386.70
80th	32.56	70.69	286.6	357.29
100th	31.40	39.86	289.2	329.06

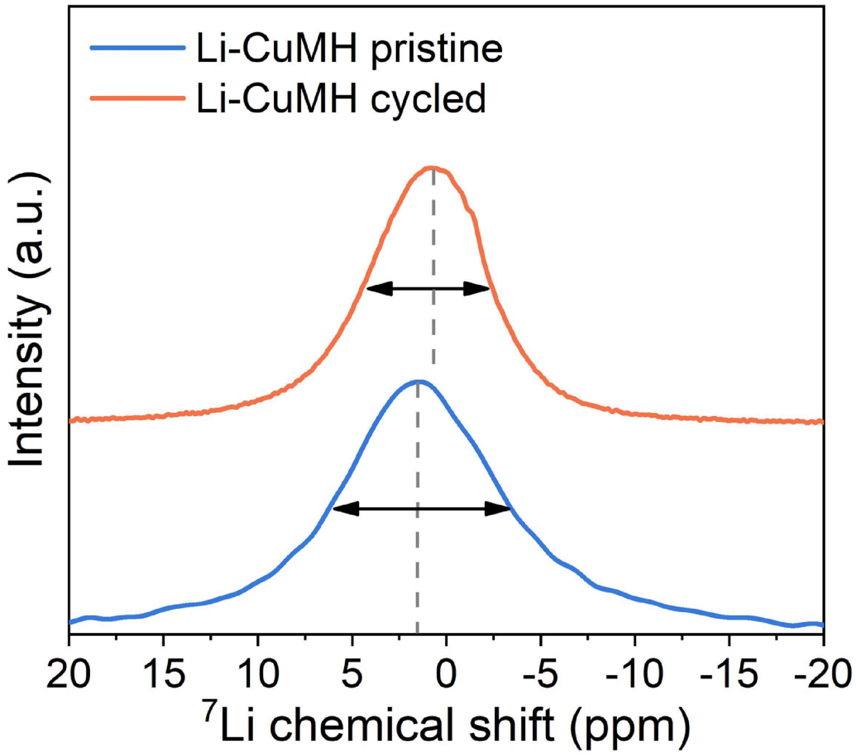

Figure S33. ^{7}Li ssMAS NMR spectra of the Li-CuMH SSE before and after cycling.

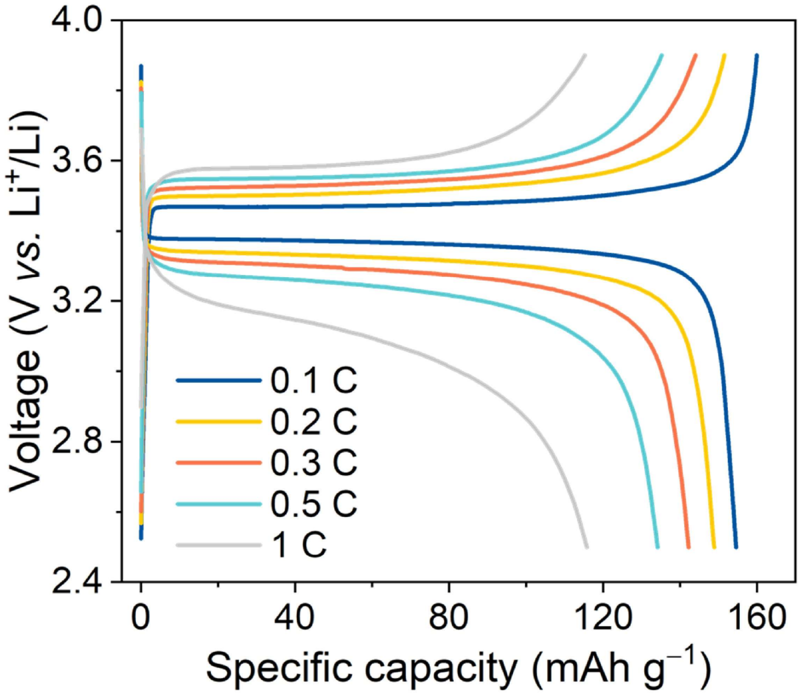

Figure S34. Charge/discharge profiles of the LFP/Li-CuMH SSE/Li battery at various current rates (1 C=170 mAh g^{-1}).

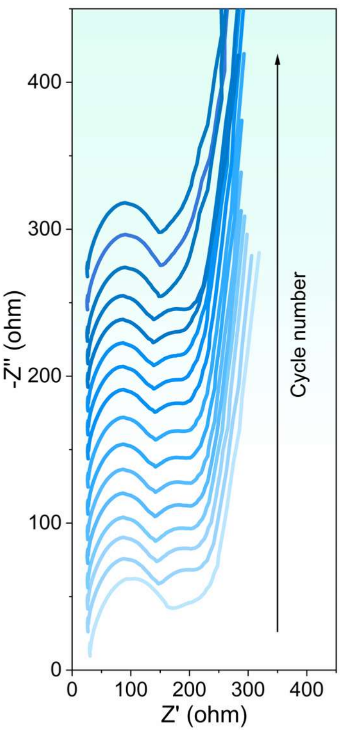

Figure S35. Nyquist plots of the LFP/Li-CuMH SSE/Li full cell before and during 150 cycles. The curves from bottom to up are the 0th, 10th, 20th, 30th, 40th, 50th, 60th, 70th, 80th, 90th, 100th, 110th, 120th, 130th, 140th, 150th cycle, respectively.

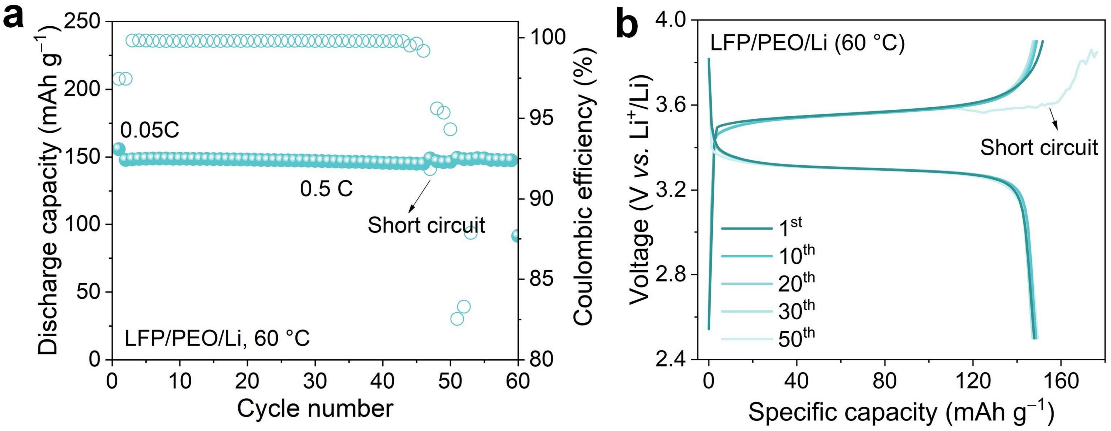

Figure S36. The long-term cycling of LFP/PEO electrolyte/Li full batteries, in which the EO: Li was 10:1. (a) The specific discharge capacity of LFP/PEO electrolyte/Li full batteries during 60 cycles at 0.5 C and 60 °C. (b) The charge/discharge curves of LFP/PEO electrolyte/Li full batteries at 1st, 20th, 30th, 40th, and 50th cycles at 0.5 C and 60 °C, respectively.

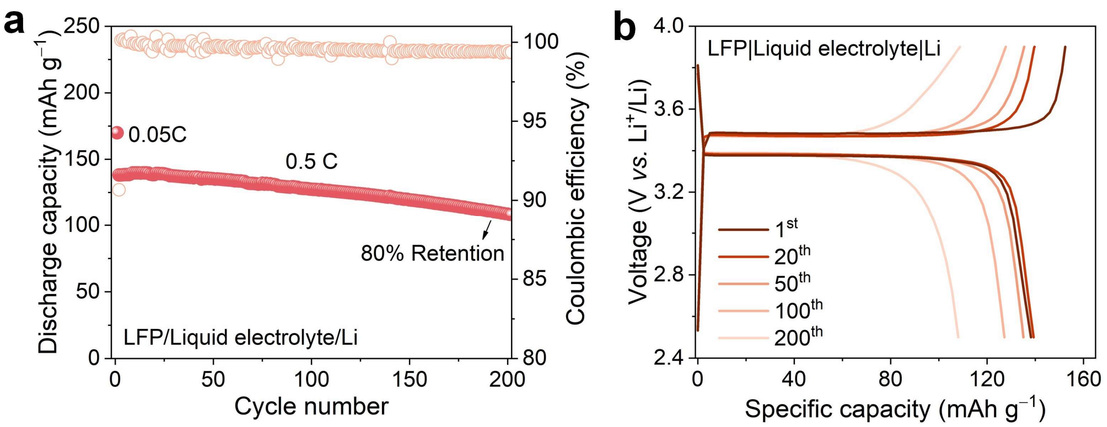

Figure S37. Long-term cycling performance of LFP/liquid electrolyte/Li full batteries, wherein liquid electrolyte was 1M LiTFSI in DOL/DME. a) Cycling stability of the LFP/liquid electrolyte/Li full cell during 200 cycles at 0.5 C and room temperature. b) Charge/discharge profiles of the LFP/liquid electrolyte/Li full cell at 1st, 20th, 50th, 100th, and 200th cycles at 0.5 C and room temperature, respectively.

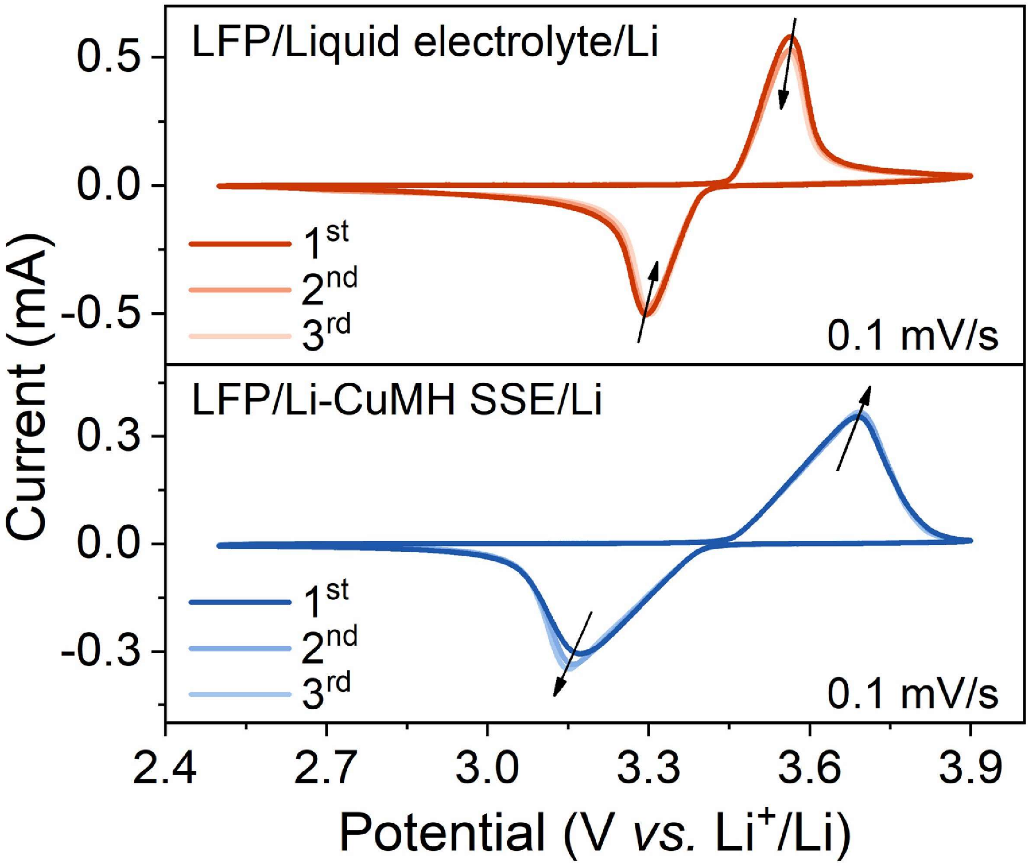

Figure S38. CV profiles of the LFP/Li full batteries with the liquid electrolyte (the upper panel) and Li-CuMH SSE (the bottom panel) at 0.1 mV s^{-1}.

Table S5. Comparision of electrochemical performance of LFP/Li batteries with the Li-CuMH SSE, liquid electrolyte and solid polymer PEO electrolyte. This table refers to Fig. 5g.

Electrolyte	Li-CuMH SSE	Liquid electrolyte	PEO SPE (60 °C)
Specific capacity (mAh g^{-1}, 1st cycle）	139.5	138.2	147.8
Coulombic efficiency（%, 1st cycle）	92.3	90.7	97.4
Lifetime	573 cycles	200 cycles	60 cycles
Cyclic stability	Good	Poor	Good
Thermal stability	Good	Poor	Good

Lifetime: The end-of-life criterion is defined as 80% of capacity retention or short circuit [10].

Liquid electrolyte: 1M LiTFSI in DOL/DME.

SPE: solid polymer electrolyte.

Table S6. Comparison of electrochemical performance of LFP/Li batteries with the Li-CuMH SSE and SSEs previously reported in the literatures.

Electrolyte	Type	Current density	Cathode mass loading (mg cm^{-2})	Capacity retention	Ref.
Li-Cu-CNF	Cellulose	0.1 C (RT)	—	200 cycles, 94%	[3]
PEO/Li_2S_6	PEO-based	0.1 mA cm^{-2} (50 °C)	3~5	700 cycles, 89.2%	[7]
HKUST-1	MOF-PTFE	1 C (50 °C)	2	500 cycles,	[8]

				75%	
CD-COF-Li	COF-based	0.1 C (RT)	2	100 cycles, 91%	[9]
LLZO/PEGMEA	Asymmetric layers	0.2 C (55 °C)	2	120 cycles, 94.5%	[11]
LZONs/PEO	Solid-polymer-solid	0.1 C (RT)	1.4	1500 cycles, 70%	[12]
Li-RCC1-ClO_4	Organic ionic cage	1 C (RT)	1	750 cycles, 88.2%	[13]
Li-CuMH SSE	2D lamellar	0.5 C (25 °C)	4	200 cycles, 90%<br>573 cycles, 80%	This work

RT: Room temperature.

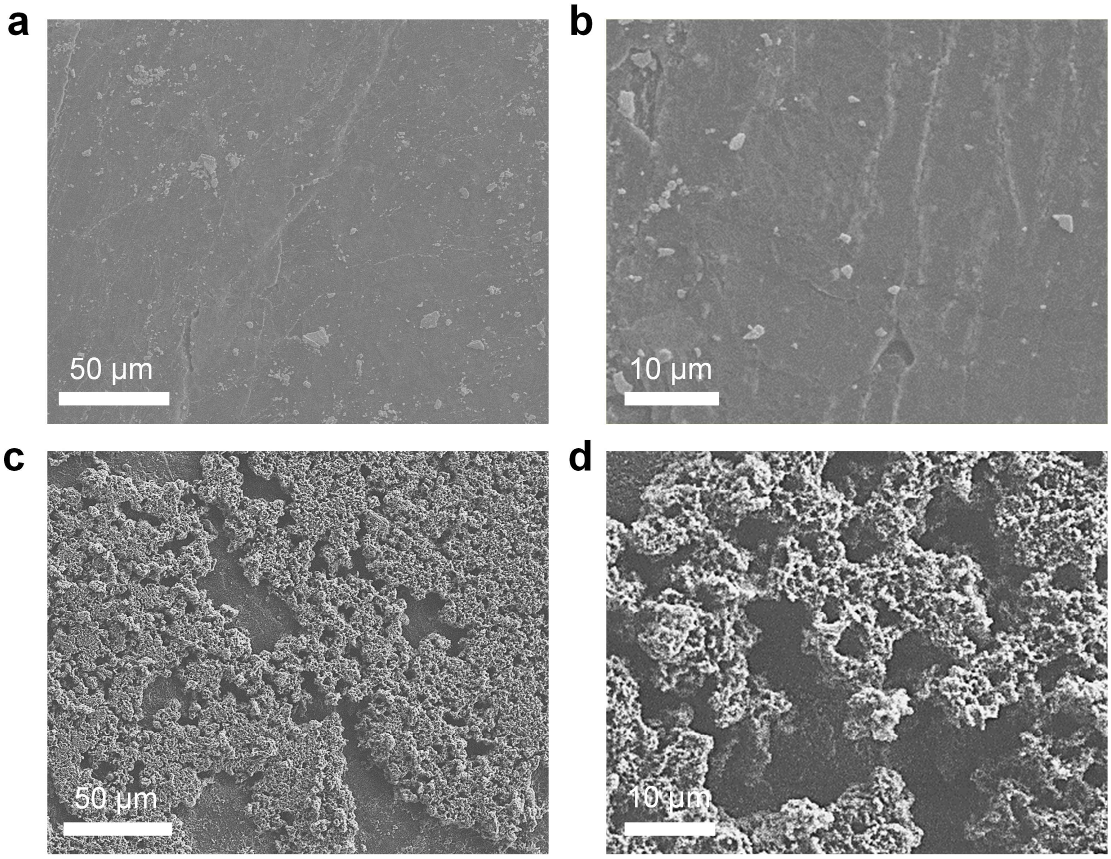

Figure S39. SEM images of Li metal anodes disassembled from Li/Li symmetric cells with a-b) Li-CuMH SSE and c-d) organic liquid electrolyte after 200 cycles, respectively.

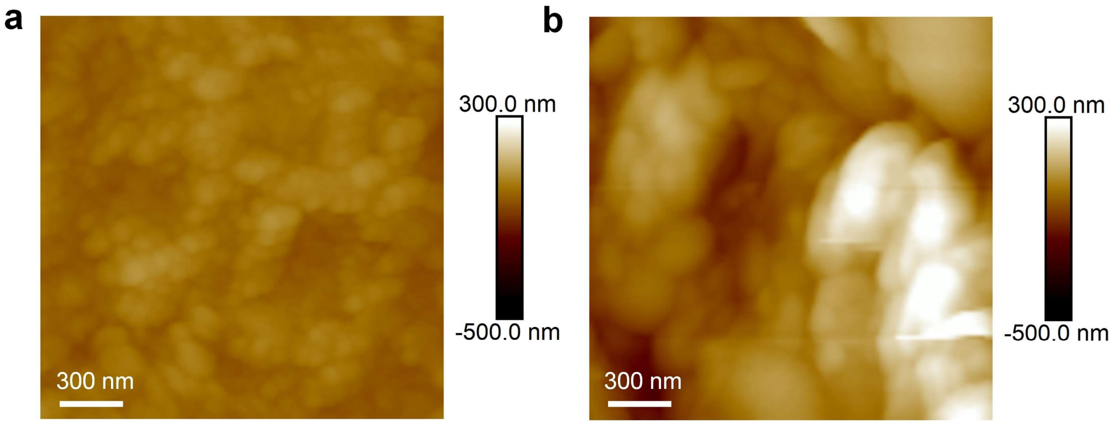

Figure S40. 2D AFM images of SEI layers on Li metal anodes disassembled from Li/Li symmetric cells with a) Li-CuMH SSE and b) organic liquid electrolyte after 200 cycles.

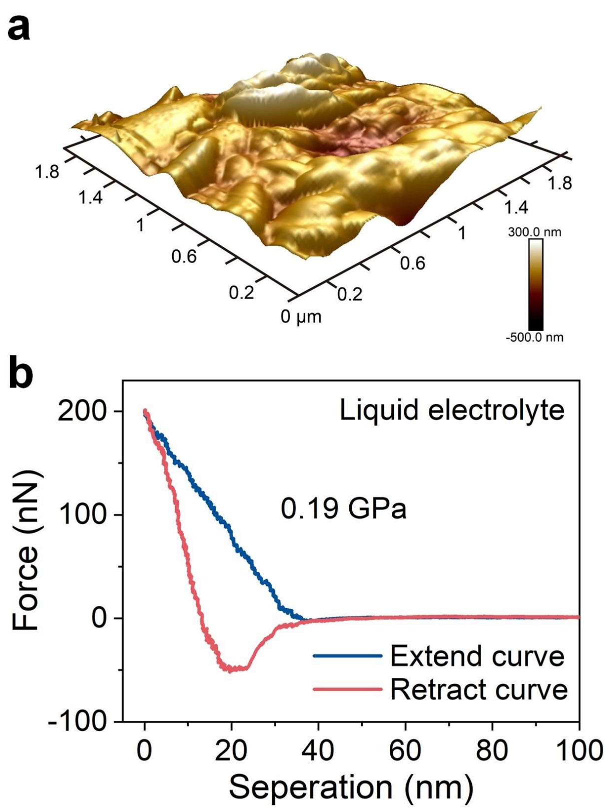

Figure S41. a) 3D AFM image and b) the corresponding force-separation curve of Li anode disassembled from Li/Li symmetric cell with liquid electrolyte.

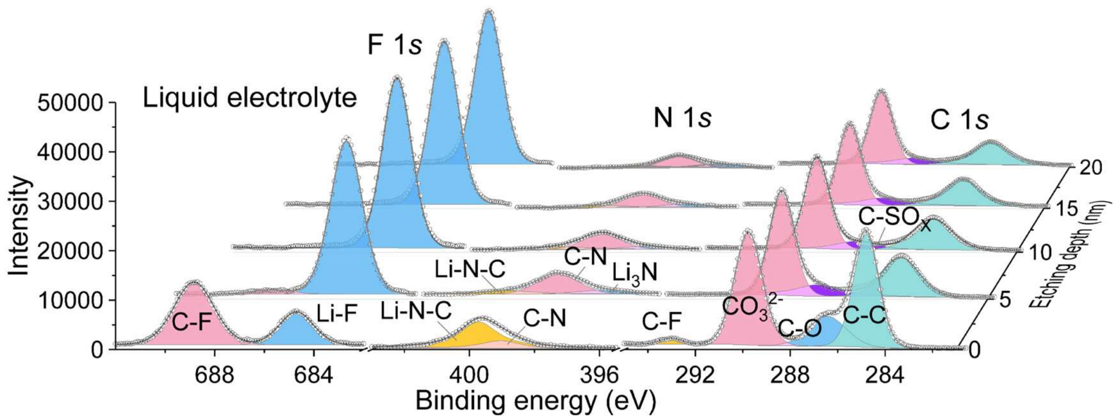

Figure S42. F 1*s*, N 1*s* and C 1*s* in-depth XPS spectra of SEI layers on Li anodes disassembled from Li/Li symmetric cells with liquid electrolyte. The raw data and fitted data plots are shown as gray hollow points and grey solid lines, respectively.

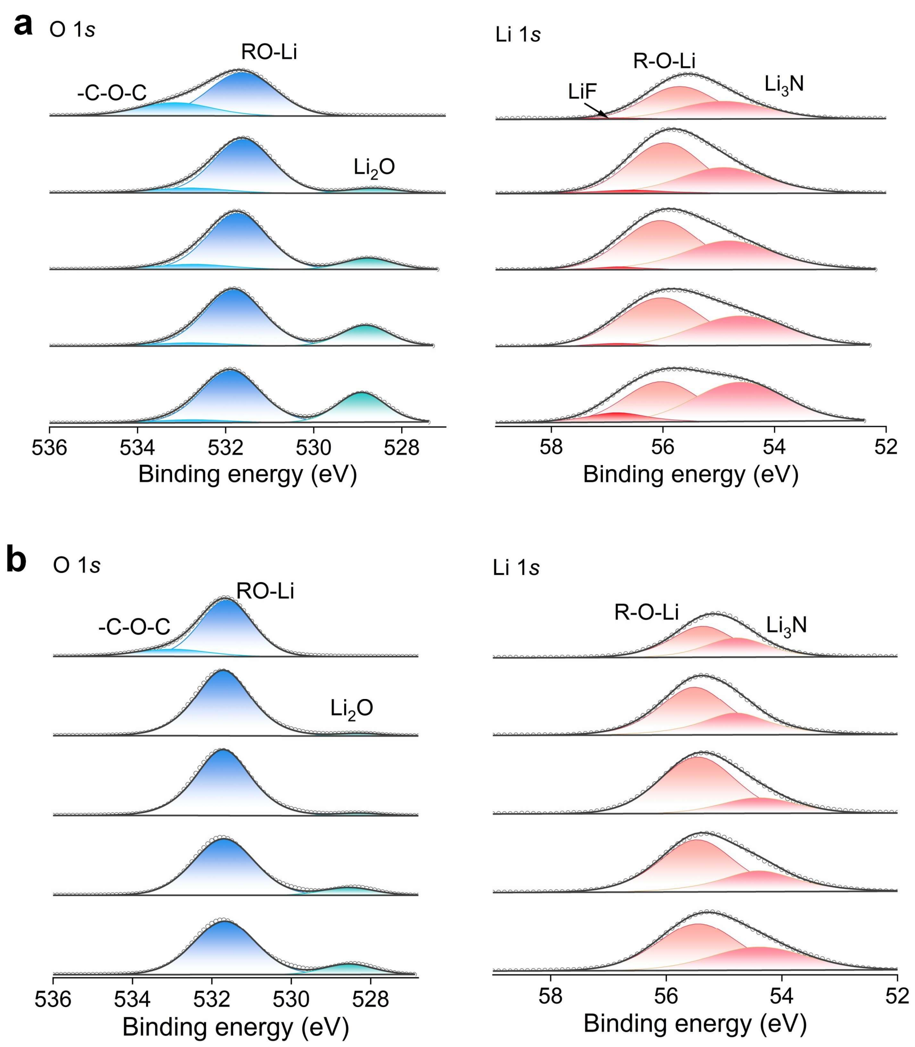

Figure S43. O 1*s* and Li 1*s* in-depth XPS spectra of SEI layers on the surface of Li metal anodes disassembled from Li/Li symmetric cells with a) Li-CuMH SSE and b) organic liquid electrolyte. The raw data and fitted data plots are shown as gray hollow points and grey solid lines, respectively.

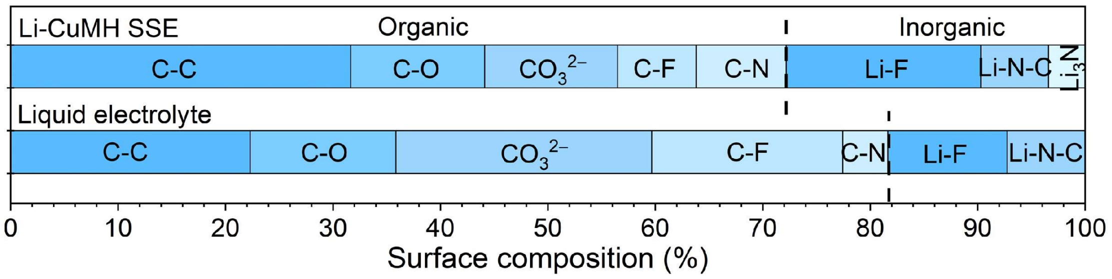

Figure S44. Quantitative surface composition of SEI layers on Li anodes disassembled from Li/Li symmetric cells with Li-CuMH SSE and liquid electrolyte.

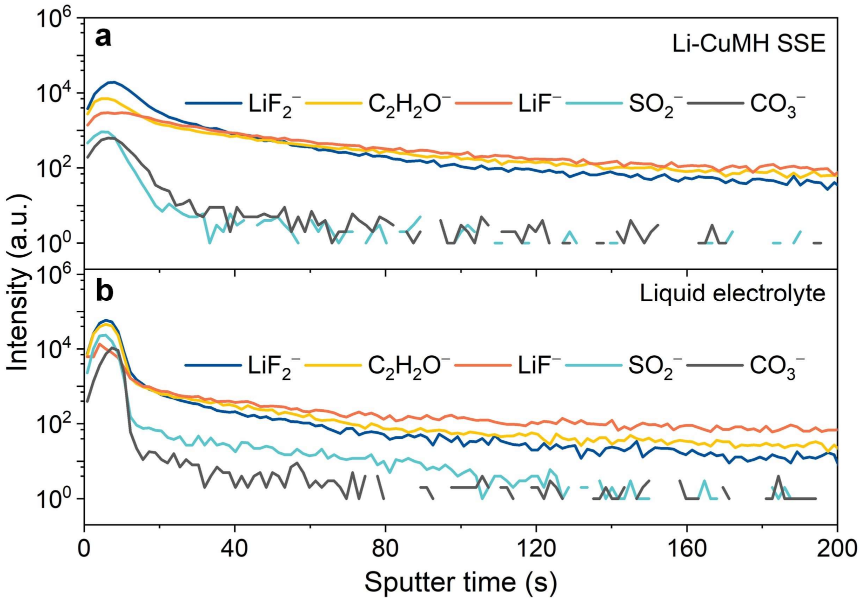

Figure S45. TOF-SIMS depth profiles of the species LiF_2^-, $C_2H_2O^-$, LiF^-, SO_2^- and CO_3^- species of SEI layers on Li metal anodes disassembled from Li/Li symmetric cells with a) Li-CuMH SSE and b) organic liquid electrolyte.

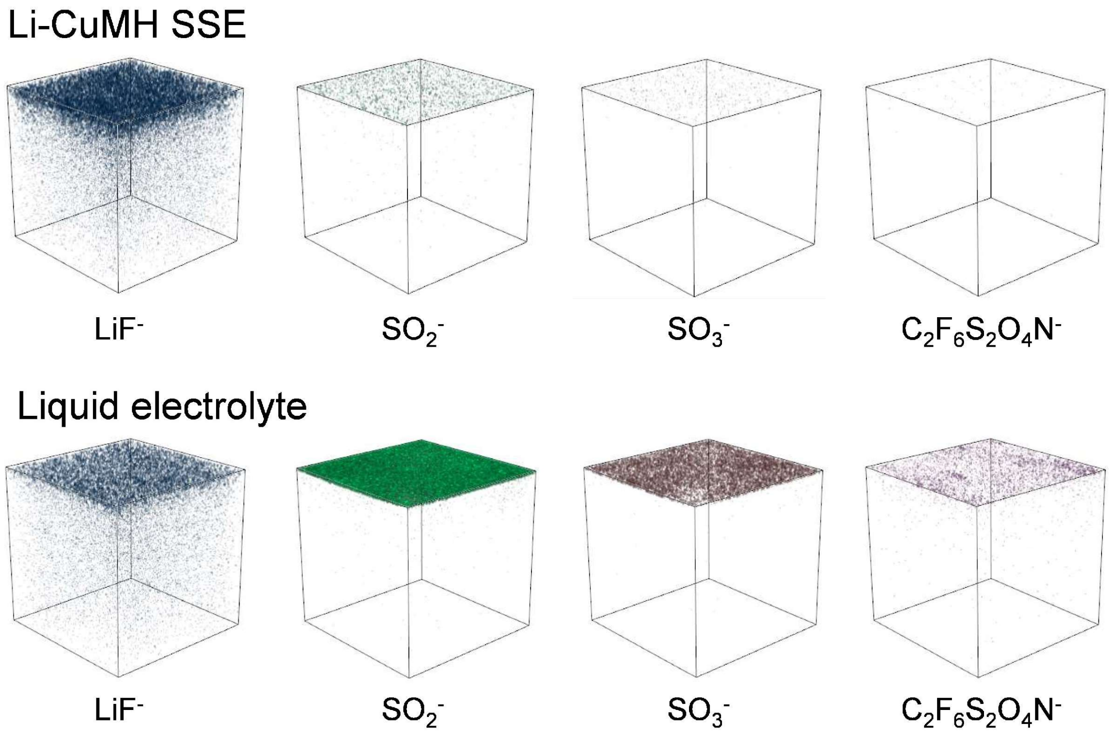

Figure S46. TOF-SIMS 3D reconstruction of LiF^-, SO_2^-, SO_3^- and $C_2F_6S_2O_4N^-$ species of SEI layers on Li anodes disassembled from Li/Li symmetric cells with Li-CuMH SSE and liquid electrolyte.

References in supplementary materials

[1] Malek, K., Vala, M., Kozłowski, H. & Proniewicz, L.M. Experimental and theoretical NMR study of selected oxocarboxylic acid oximes. *Magn. Reson. Chem.* **42,** 23-29 (2003).

[2] Salem, A.A., Mossa, H.A. & Barsoum, B.N. Quantitative determinations of levofloxacin and rifampicin in pharmaceutical and urine samples using nuclear magnetic resonance spectroscopy. *Spectrochim. Acta. A Mol. Biomol. Spectrosc.* **62,** 466-472 (2005).

[3] Yang, C.P. et al. Copper-coordinated cellulose ion conductors for solid-state batteries. *Nature* **598,** 590-596 (2021).

[4] Su, Y. et al. Rational design of a topological polymeric solid electrolyte for high-performance all-solid-state alkali metal batteries. *Nat. Commun.* **13,** 4181 (2022).

[5] Wang, Y. et al. Solid-state rigid-rod polymer composite electrolytes with nanocrystalline lithium ion pathways. *Nat. Mater.* **20,** 1255-1263 (2021).

[6] Xu, B. et al. Interfacial chemistry enables stable cycling of all-solid-state Li metal batteries at high current densities. *J. Am. Chem. Soc.* **143,** 6542-6550 (2021).

[7] Fang, R. et al. Li_2S_6-integrated PEO-based polymer electrolytes for all-solid-state lithium-metal batteries. *Angew. Chem. Int. Ed.* **60,** 17701-17706 (2021).

[8] Shen, L. et al. Creating lithium-ion electrolytes with biomimetic ionic channels in metal-organic frameworks. *Adv. Mater.* **30,** 1707476 (2018).

[9] Wang, X.-X. et al. An integrated solid-state lithium-oxygen battery with highly stable anionic covalent organic frameworks electrolyte. *Chem* **9,** 394-410 (2022).

[10] Chen, Z. et al. Highly stable quasi-solid-state lithium metal batteries: Reinforced $Li_{1.3}Al_{0.3}Ti_{1.7}(PO_4)_3$/Li interface by a protection interlayer. *Adv. Energy Mater.* **11,** 2101339 (2021).

[11] Duan, H. et al. Dendrite-free Li-metal battery enabled by a thin asymmetric solid electrolyte with engineered layers. *J. Am. Chem. Soc.* **140,** 82-85 (2018).

[12] Ma, J. et al. Constructing a highly efficient "solid-polymer-solid" elastic ion transport network in cathodes activates the room temperature performance of all-solid-state lithium batteries. *Energy Environ. Sci.* **15,** 1503-1511 (2022).

[13] Li, J. et al. Room temperature all-solid-state lithium batteries based on a soluble organic cage ionic conductor. *Nat. Commun.* **13,** 2031 (2022).
