# Supplementary material for: Self-assembled hydrated copper coordination compounds as ionic conductors for room temperature solid-state batteries
Source: Nat Commun. 2024 Feb 5;15:1056. doi: 10.1038/s41467-024-45372-2 (PMC10844207; doi:10.1038/s41467-024-45372-2)
Supplement: Supplementary file 3 — Description of Additional Supplementary Files [file 41467_2024_45372_MOESM3_ESM.pdf]

Description of Additional Supplementary Data files

Supplementary Data 1

350K-MD.txt: Mean square displacement (MSD) data of Li, H, O, C and Cu in Li-CuMH at 350 K, respectively.

450K-MD.txt: Mean square displacement (MSD) data of Li, H, O, C and Cu in Li-CuMH at 450 K, respectively.

550K-MD.txt: Mean square displacement (MSD) data of Li, H, O, C and Cu in Li-CuMH at 550 K, respectively.

650K-MD.txt: Mean square displacement (MSD) data of Li, H, O, C and Cu in Li-CuMH at 650 K, respectively.

750K-MD.txt: Mean square displacement (MSD) data of Li, H, O, C and Cu in Li-CuMH at 750 K, respectively.

CuMH.txt: Atomic coordinates of the CuMH model.

Li-CuMH.txt: Atomic coordinates of the Li-CuMH model.
